# Supplementary material for: Ancestry-Dependent Enrichment of Deleterious Homozygotes in Runs of Homozygosity
Source: Am J Hum Genet. 2019 Sep 19;105(4):747–62. doi: 10.1016/j.ajhg.2019.08.011 (PMC6817522; doi:10.1016/j.ajhg.2019.08.011)
Supplement: Document S1. Figures S1–S10, Tables S1–S9, S11–S14, S16, and S17, Supplemental Material and Methods, and Supplemental Acknowledgments [file mmc1.pdf]

The American Journal of Human Genetics, Volume *105*

Supplemental Data

Ancestry-Dependent Enrichment of Deleterious Homozygotes in Runs of Homozygosity

Zachary A. Szpiech, Angel C.Y. Mak, Marquitta J. White, Donglei Hu, Celeste Eng, Esteban G. Burchard, and Ryan D. Hernandez

Supplemental Data

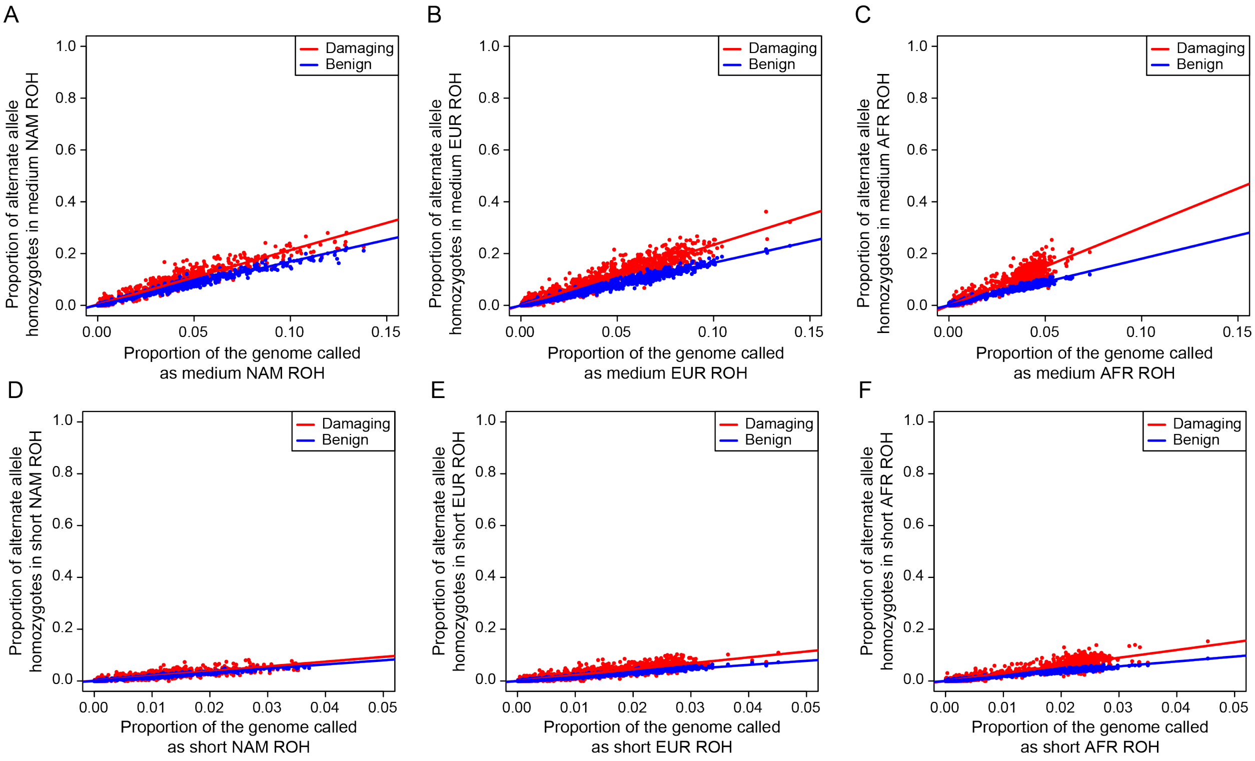

Figure S1. Deleterious versus benign homozygotes by ancestry for medium and small ROH.

The proportion of damaging (red) and benign (blue) homozygotes falling in ROH comprised of different ancestral haplotypes and size classes: (A) medium NAM ROH, (B) medium EUR ROH, (C) medium AFR ROH, (D) short NAM ROH, (E) short EUR ROH, and (F) short AFR ROH. EUR – European, AFR – African, and NAM – Native American.

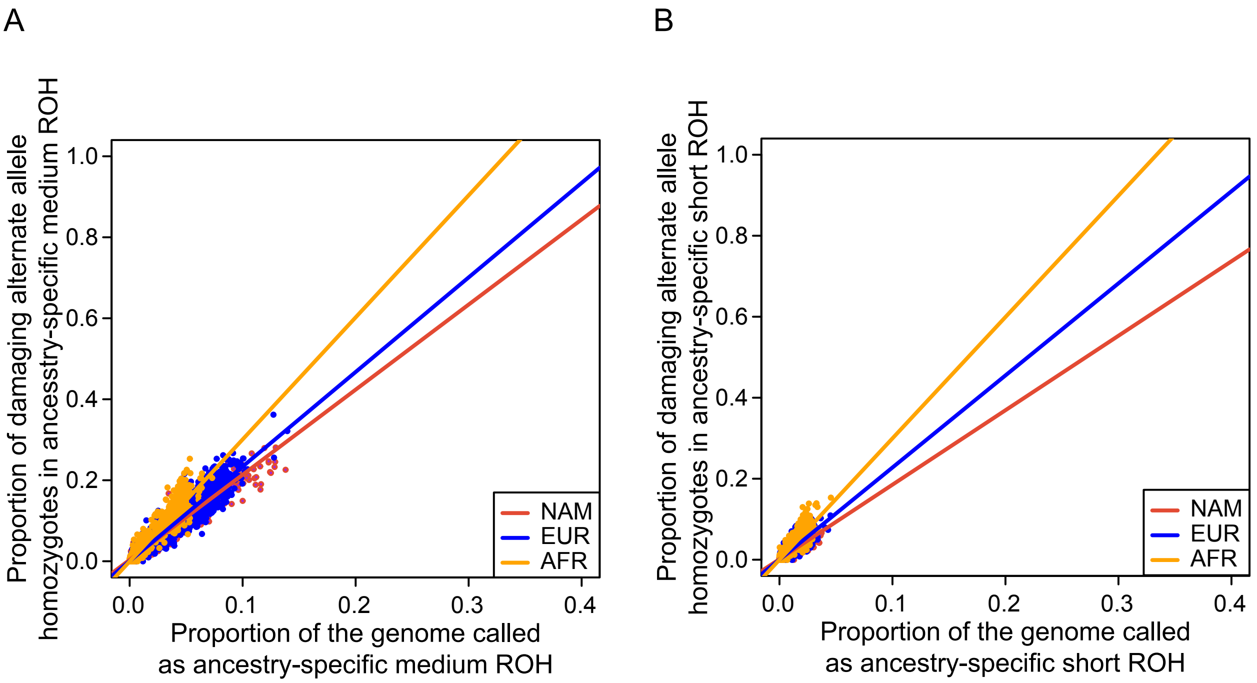

Figure S2. Comparison of deleterious homozygotes between ancestries for medium and small ROH.

A direct comparison of the proportion of damaging homozygotes falling in ROH comprised of different ancestral haplotypes for (A) medium ROH and (B) short ROH. EUR – European, colored blue; AFR – African, colored orange; and NAM – Native American, colored red.

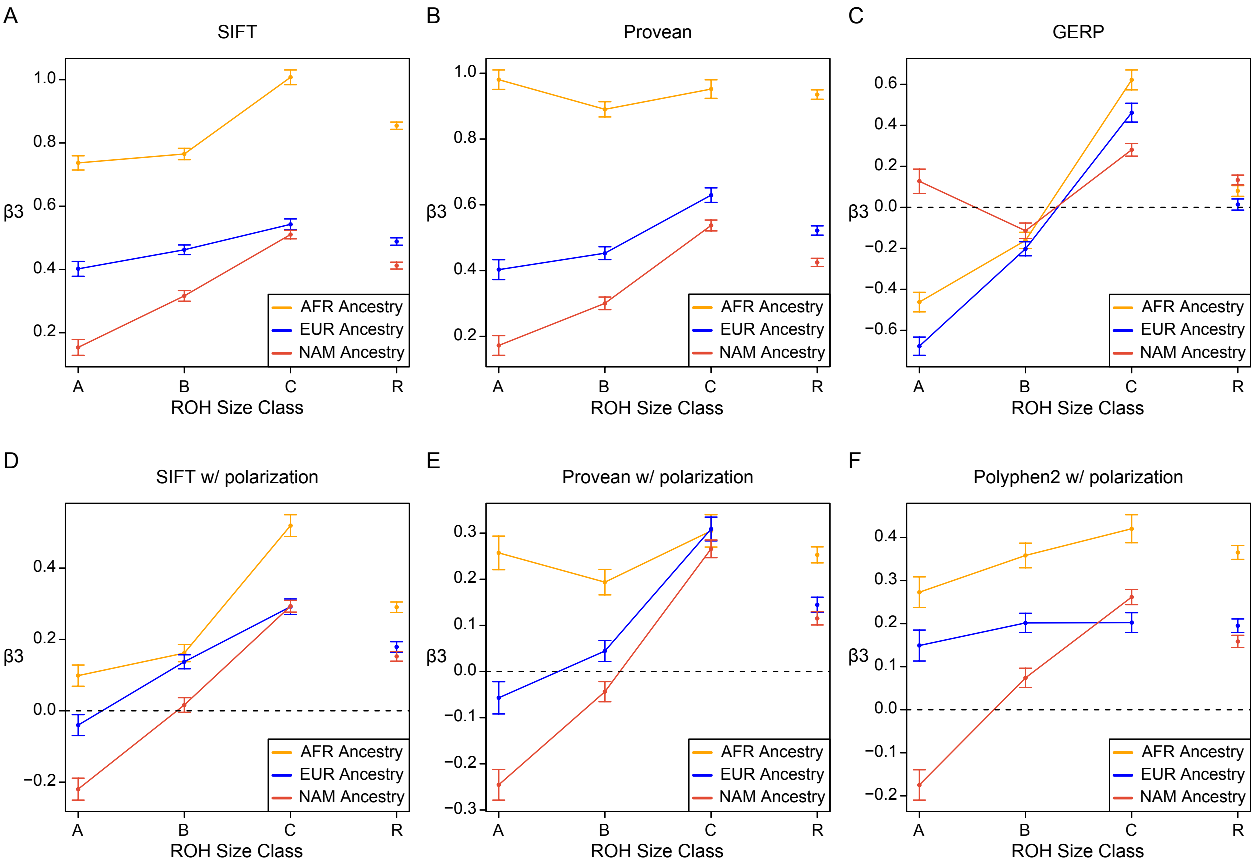

Figure S3. Regression coefficients for analyses with other deleteriousness classifications.

The difference in slopes (β_3 coefficients) between deleterious and benign categories across ROH size classes from re-analyses of the data using different deleteriousness classification schemes. (A) SIFT, (B) Provean, (C) GERP, (D) SIFT only with derived alleles, (E) Provean only with derived alleles, (F) Polyphen 2 only with derived alleles. ROH size classes: A – short, B – medium, C – long, R – all sizes. EUR – European, colored blue; AFR – African, colored orange; and NAM – Native American, colored red.

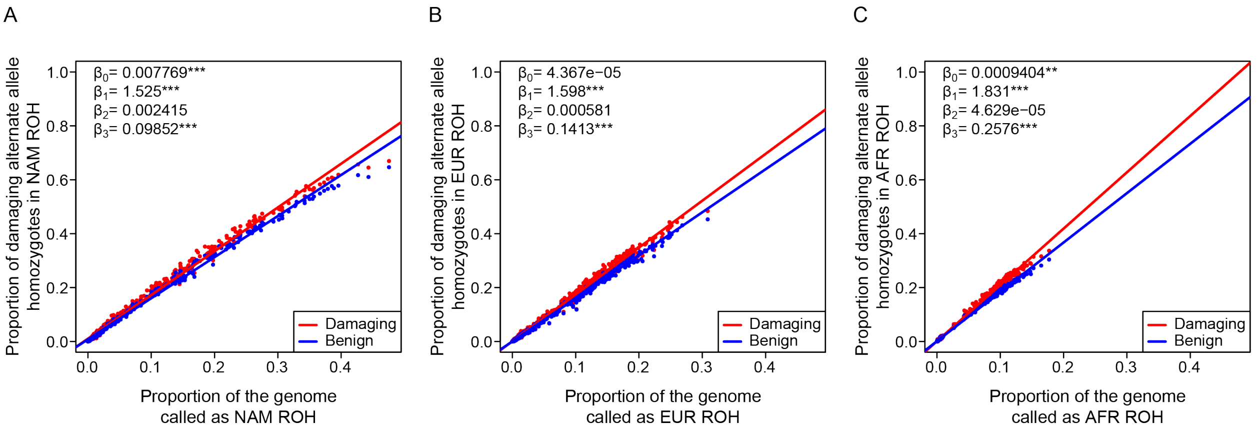

Figure S4. Replication of findings using 1000 genomes data.
The identical analysis from Fig 4A-C except using the six admixed populations from the 1000 Genomes Project and CADD scores. (A) Native American ancestry, (B) European Ancestry, (C) African Ancestry.

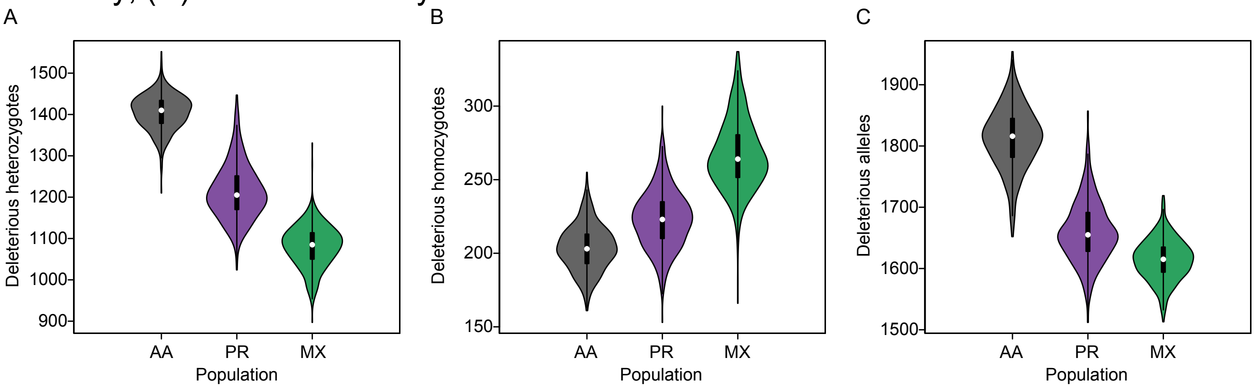

Figure S5. The distribution of polarized Polyphen2 deleterious alleles across populations.
The number of (A) deleterious heterozygotes, (B) deleterious homozygotes, and (C) total deleterious alleles per individual using polarized Polyphen2 classifications. AA – African American, PR – Puerto Rican, MX – Mexican American.

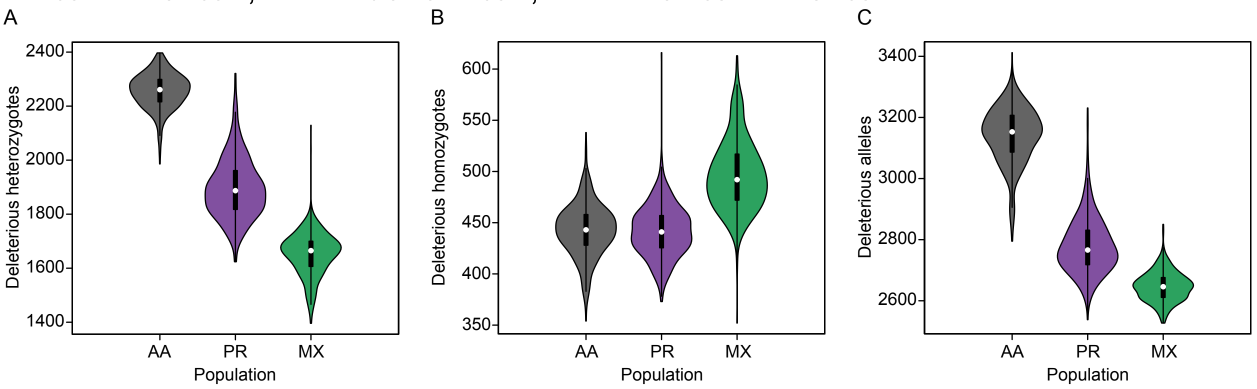

Figure S6. The distribution of SIFT deleterious alleles across populations.
The number of (A) deleterious heterozygotes, (B) deleterious homozygotes, and (C) total deleterious alleles per individual using SIFT classifications. AA – African American, PR – Puerto Rican, MX – Mexican American.

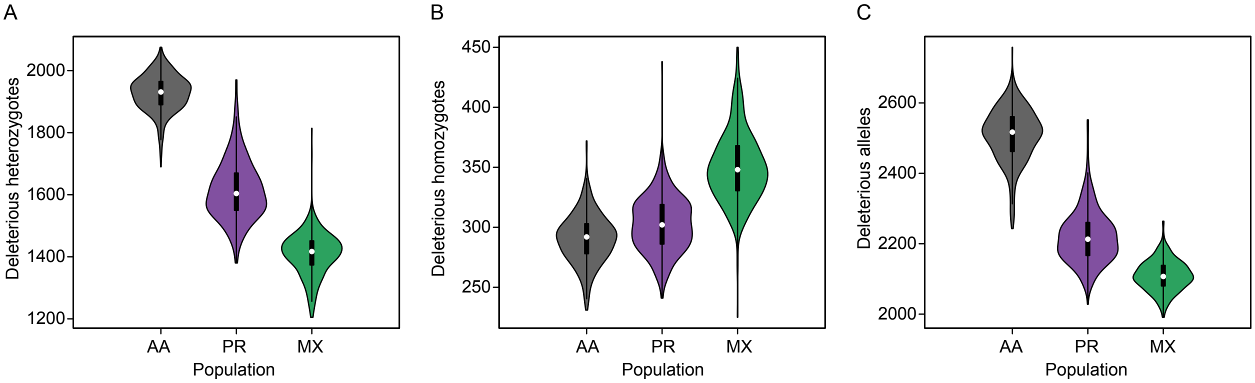

Figure S7. The distribution of polarized SIFT deleterious alleles across populations.
The number of (A) deleterious heterozygotes, (B) deleterious homozygotes, and (C) total deleterious alleles per individual using polarized SIFT classifications. AA – African American, PR – Puerto Rican, MX – Mexican American.

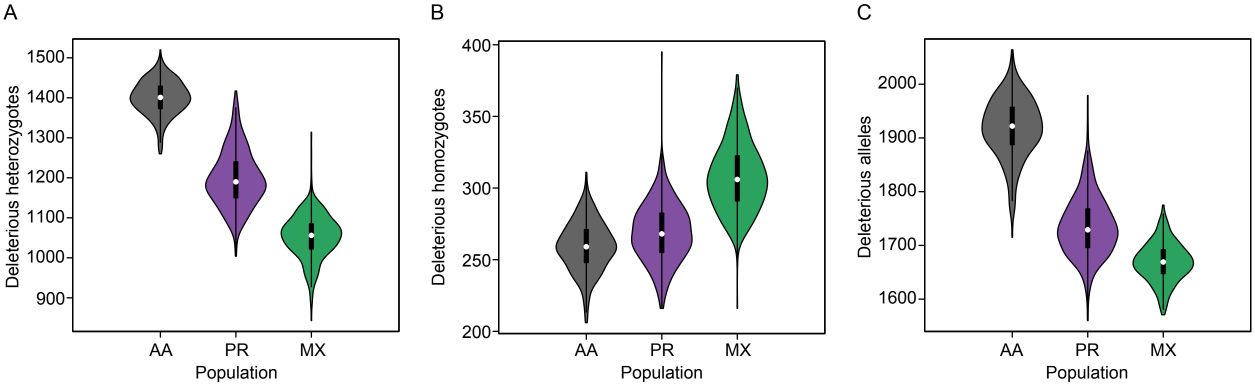

Figure S8. The distribution of Provean deleterious alleles across populations.
The number of (A) deleterious heterozygotes, (B) deleterious homozygotes, and (C) total deleterious alleles per individual using Provean classifications. AA – African American, PR – Puerto Rican, MX – Mexican American.

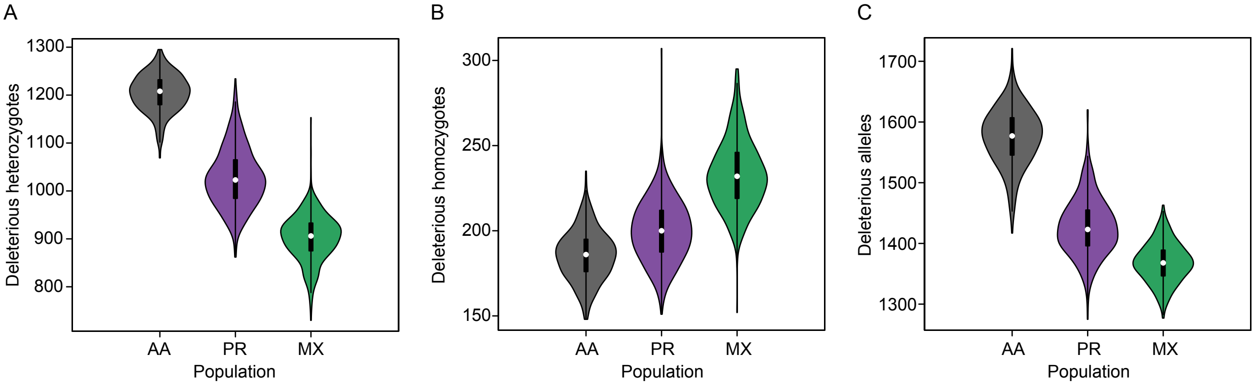

Figure S9. The distribution of polarized Provean deleterious alleles across populations.
The number of (A) deleterious heterozygotes, (B) deleterious homozygotes, and (C) total deleterious alleles per individual using polarized Provean classifications. AA – African American, PR – Puerto Rican, MX – Mexican American.

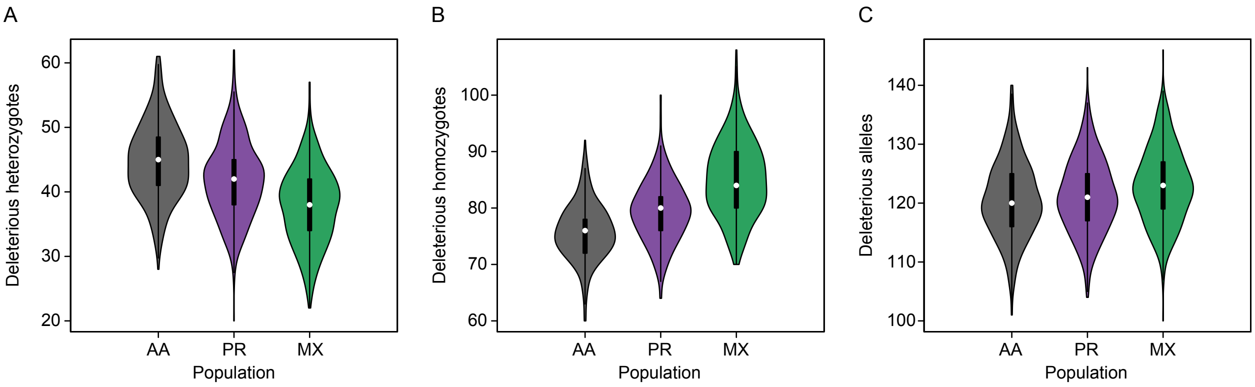

Figure S10. The distribution of GERP deleterious alleles across populations. The number of (A) deleterious heterozygotes, (B) deleterious homozygotes, and (C) total deleterious alleles per individual using GERP classifications. AA – African American, PR – Puerto Rican, MX – Mexican American.

Table S1. Regression coefficients inferred for the analyses shown in Figure 4 with p-values in parentheses.

ROH Class	β_0 (p-value)	β_1 (p-value)	β_2 (p-value)	β_3 (p-value)
All	***3.122×10^{-2} ($< 2 \times 10^{-16}$)	***1.460 ($< 2 \times 10^{-16}$)	***0.180 ($< 2 \times 10^{-16}$)	1.807×10^{-2} (0.0671)
Long	1.059×10^{-3} (0.508)	***1.429 ($< 2 \times 10^{-16}$)	***5.335×10^{-2} ($< 2 \times 10^{-16}$)	***0.229 ($< 2 \times 10^{-16}$)
Medium	***8.510×10^{-3} ($< 3.86 \times 10^{-5}$)	***1.584 ($< 2 \times 10^{-16}$)	***7.012×10^{-2} ($< 2 \times 10^{-16}$)	*5.695×10^{-2} (0.0173)
Short	***1.265×10^{-2} (4.61×10^{-7})	***1.424 ($< 2 \times 10^{-16}$)	***4.810×10^{-2} ($< 2 \times 10^{-16}$)	***-0.428 (1.10×10^{-8})

* $p < 0.05$, ** $p < 0.01$, *** $p < 0.001$.

Table S2. Regression coefficients inferred for the analyses shown in Figure 5 and Figure S1 with p-values in parentheses.

ROH Class	Ancestral Haplotype	β_0 (p-value)	β_1 (p-value)	β_2 (p-value)	β_3 (p-value)
All	NAM	***3.455×10^{-3} (3.18×10^{-4})	***1.594 ($< 2 \times 10^{-16}$)	***7.369×10^{-3} (5.87×10^{-8})	***0.510 ($< 2 \times 10^{-16}$)
	EUR	-1.205×10^{-3} (0.2081)	***1.546 ($< 2 \times 10^{-16}$)	2.497×10^{-3} (6.52×10^{-2})	***0.648 ($< 2 \times 10^{-16}$)
	AFR	*1.308×10^{-3} (3.11×10^{-2})	***1.743 ($< 2 \times 10^{-16}$)	-1.210×10^{-3} (0.1587)	***1.214 ($< 2 \times 10^{-16}$)
Long	NAM	**1.989×10^{-3} (3.38×10^{-3})	***1.545 ($< 2 \times 10^{-16}$)	***4.192×10^{-3} (1.28×10^{-5})	***0.604 ($< 2 \times 10^{-16}$)
	EUR	-3.026×10^{-4} (0.623)	***1.403 ($< 2 \times 10^{-16}$)	1.260×10^{-3} (0.148)	***0.624 ($< 2 \times 10^{-16}$)
	AFR	5.324×10^{-4} (0.286)	***1.610 ($< 2 \times 10^{-16}$)	1.134×10^{-4} (0.872)	***1.265 ($< 2 \times 10^{-16}$)
Medium	NAM	*1.237×10^{-3} (1.661×10^{-2})	***1.679 ($< 2 \times 10^{-16}$)	**2.325×10^{-3} (1.46×10^{-3})	***0.421 ($< 2 \times 10^{-16}$)
	EUR	-5.614×10^{-4} (0.340)	***1.651 ($< 2 \times 10^{-16}$)	8.918×10^{-4} (0.284)	***0.684 ($< 2 \times 10^{-16}$)
	AFR	4.961×10^{-4} (0.246)	***1.797 ($< 2 \times 10^{-16}$)	-1.001×10^{-3} (0.0979)	***1.217 ($< 2 \times 10^{-16}$)
Short	NAM	2.711×10^{-4} (0.259)	***1.594 ($< 2 \times 10^{-16}$)	**1.073×10^{-3} (1.61×10^{-3})	***0.244 (1.27×10^{-13})
	EUR	-1.125×10^{-4} (0.720)	***1.665 ($< 2 \times 10^{-16}$)	4.023×10^{-4} (0.364)	***0.609 ($< 2 \times 10^{-16}$)
	AFR	2.349×10^{-4} (0.430)	***1.881 ($< 2 \times 10^{-16}$)	-2.568×10^{-4} (0.542)	***1.116 ($< 2 \times 10^{-16}$)

* $p < 0.05$, ** $p < 0.01$, *** $p < 0.001$.

Table S3. Regression coefficients inferred for the analyses shown in Figure 6 and Figure S2 with p-values in parentheses.

ROH Class	β_0 (p-value)	β_1 (p-value)	β_2 (p-value)	β_3 (p-value)	β_4 (p-value)	β_5 (p-value)
All	9.861×10^{-5} (0.922)	***2.957 ($< 2 \times 10^{-16}$)	1.193×10^{-3} (0.431)	***-1.072×10^{-2} (1.87×10^{-12})	***-0.763 ($< 2 \times 10^{-16}$)	***-0.852 ($< 2 \times 10^{-16}$)
Long	5.002×10^{-4} (0.449)	***2.878 ($< 2 \times 10^{-16}$)	4.477×10^{-4} (0.661)	***5.453×10^{-3} (7.11×10^{-8})	***-0.852 ($< 2 \times 10^{-16}$)	***-0.727 ($< 2 \times 10^{-16}$)
Medium	-4.804×10^{-4} (0.443)	***3.013 ($< 2 \times 10^{-16}$)	8.098×10^{-4} (0.387)	***4.002×10^{-3} (2.38×10^{-5})	***-0.678 ($< 2 \times 10^{-16}$)	***-0.912 ($< 2 \times 10^{-16}$)
Short	-2.196×10^{-5} (0.953)	***2.996 ($< 2 \times 10^{-16}$)	3.114×10^{-4} (0.571)	*1.351×10^{-3} (0.0149)	***-0.723 ($< 2 \times 10^{-16}$)	***-1.158 ($< 2 \times 10^{-16}$)

* p < 0.05, ** p < 0.01, *** p < 0.001.

Table S4. Regression coefficients inferred for the analyses comparing deleterious homozygotes in high-pLI versus low-pLI gene regions with p-values in parentheses.

ROH Class	β_0 (p-value)	β_1 (p-value)	β_2 (p-value)	β_3 (p-value)
All	***0.209 ($< 2 \times 10^{-16}$)	***1.463 ($< 2 \times 10^{-16}$)	***-9.924×10^{-5} (0.992)	*7.243×10^{-2} (0.0253)
Long	***5.280×10^{-2} ($< 2 \times 10^{-16}$)	***1.637 ($< 2 \times 10^{-16}$)	3.482×10^{-3} (0.634)	9.639×10^{-2} (0.0960)
Medium	***7.894×10^{-2} ($< 2 \times 10^{-16}$)	***1.641 ($< 2 \times 10^{-16}$)	5.712×10^{-2} (0.552)	-2.262×10^{-2} (0.774)
Short	***5.869×10^{-2} (3.10×10^{-13})	***0.975 (8.89×10^{-9})	-2.103×10^{-2} (0.0636)	*0.502 (0.0359)

* p < 0.05, ** p < 0.01, *** p < 0.001.

Table S5. Regression coefficients inferred for the analysis of deleterious homozygotes in high-pLI versus low-pLI genes with p-values in parentheses.

ROH Class	Ancestral Haplotype	β_0 (p-value)	β_1 (p-value)	β_2 (p-value)	β_3 (p-value)
All	NAM	***1.048×10^{-2} (2.54×10^{-6})	***2.087 ($< 2 \times 10^{-16}$)	1.065×10^{-3} (0.735)	**0.0746 (9.030×10^{-3})
	EUR	4.995×10^{-4} (0.849)	***2.160 ($< 2 \times 10^{-16}$)	6.582×10^{-3} (0.0767)	0.0584 (0.114)
	AFR	4.160×10^{-4} (0.828)	***2.947 ($< 2 \times 10^{-16}$)	-3.183×10^{-3} (0.239)	-8.246×10^{-3} (0.852)
Long	NAM	***6.190×10^{-3} (6.360×10^{-4})	***2.134 ($< 2 \times 10^{-16}$)	3.357×10^{-4} (0.896)	*0.0973 (1.571×10^{-2})
	EUR	7.646×10^{-4} (0.680)	***1.963 ($< 2 \times 10^{-16}$)	1.824×10^{-3} (0.487)	*0.133 (2.520×10^{-2})
	AFR	6.344×10^{-4} (0.709)	***2.882 ($< 2 \times 10^{-16}$)	-2.579×10^{-4} (0.915)	0.146 (0.122)
Medium	NAM	*3.217×10^{-3} (3.730×10^{-2})	***2.087 ($< 2 \times 10^{-16}$)	2.060×10^{-3} (0.346)	0.0595 (0.313)
	EUR	-2.446×10^{-4} (0.893)	***2.347 ($< 2 \times 10^{-16}$)	3.787×10^{-3} (0.140)	-0.0894 (0.136)
	AFR	4.961×10^{-4} (0.825)	***3.020 ($< 2 \times 10^{-16}$)	-1.287×10^{-3} (0.552)	0.0922 (0.288)
Short	NAM	1.299×10^{-3} (0.0528)	***1.786 ($< 2 \times 10^{-16}$)	-8.920×10^{-4} (0.347)	-0.0725 (0.427)
	EUR	3.451×10^{-4} (0.763)	***2.194 ($< 2 \times 10^{-16}$)	5.937×10^{-4} (0.714)	**0.304 (8.350×10^{-3})
	AFR	1.858×10^{-4} (0.851)	***2.923 ($< 2 \times 10^{-16}$)	-1.299×10^{-3} (0.354)	***-0.496 (2.260×10^{-6})

* $p < 0.05$, ** $p < 0.01$, *** $p < 0.001$.

Table S6. Regression coefficients for the analysis of deleterious homozygotes in high-pLI genes compared across ancestry backgrounds with p-values in parentheses.

ROH Class	β_0 (p-value)	β_1 (p-value)	β_2 (p-value)	β_3 (p-value)	β_4 (p-value)	β_5 (p-value)
All	-2.767×10^{-3} (0.312)	***2.938 ($< 2 \times 10^{-16}$)	*9.848×10^{-3} (1.747×10^{-2})	***1.431×10^{-2} (5.580×10^{-4})	***−0.720 ($< 2 \times 10^{-16}$)	***−0.777 ($< 2 \times 10^{-16}$)
Long	2.916×10^{-4} (0.883)	***3.030 ($< 2 \times 10^{-16}$)	2.271×10^{-3} (0.458)	*5.994×10^{-3} (4.790×10^{-2})	***−0.934 ($< 2 \times 10^{-16}$)	***−0.796 ($< 2 \times 10^{-16}$)
Medium	-1.545×10^{-3} (0.442)	***3.110 ($< 2 \times 10^{-16}$)	5.076 (0.0904)	*6.762×10^{-3} (2.570×10^{-2})	***−0.852 ($< 2 \times 10^{-16}$)	***−0.962 ($< 2 \times 10^{-16}$)
Short	-1.113×10^{-3} (0.368)	***2.427 ($< 2 \times 10^{-16}$)	2.051×10^{-3} (0.266)	1.515×10^{-3} (0.415)	0.071 (0.597)	***−0.713 (1.270×10^{-5})

* $p < 0.05$, ** $p < 0.01$, *** $p < 0.001$.

Table S7. Regression coefficients for the meta-analysis of simulation results with p-values in parentheses.

ROH Class	β_0 (p-value)	β_1 (p-value)	β_2 (p-value)	β_3 (p-value)	β_4 (p-value)	β_5 (p-value)
All	***5.781×10^{-2} ($< 2 \times 10^{-16}$)	***1.687 ($< 2 \times 10^{-16}$)	***4.209×10^{-2} ($< 2 \times 10^{-16}$)	***5.209×10^{-2} ($< 2 \times 10^{-16}$)	***−0.409 ($< 2 \times 10^{-16}$)	***−0.488 ($< 2 \times 10^{-16}$)
Long	***1.657×10^{-2} ($< 2 \times 10^{-16}$)	***1.907 ($< 2 \times 10^{-16}$)	***3.277×10^{-3} (3.950×10^{-7})	***4.892×10^{-3} (3.645×10^{-12})	***−0.386 ($< 2 \times 10^{-16}$)	***−0.446 ($< 2 \times 10^{-16}$)
Medium	***5.049×10^{-3} (6.983×10^{-7})	***1.976 ($< 2 \times 10^{-16}$)	**4.819×10^{-3} (3.246×10^{-3})	***6.103×10^{-3} (2.979×10^{-4})	***−0.390 ($< 2 \times 10^{-16}$)	***−0.473 ($< 2 \times 10^{-16}$)
Short	*2.731×10^{-3} (0.0247)	***1.969 ($< 2 \times 10^{-16}$)	2.497×10^{-3} (0.151)	-2.329×10^{-3} (0.182)	***−0.402 ($< 2 \times 10^{-16}$)	***−0.411 ($< 2 \times 10^{-16}$)

* $p < 0.05$, ** $p < 0.01$, *** $p < 0.001$.

Table S8. Top 0.1% of genes with high ROH coverage.

Gene Symbol	Description	AA	MX	PR
AC006486.9	Uncharacterized	X		
ALDOC	Aldolase, Fructose-Bisphosphate C			X
ALG2	ALG2 Alpha-1,3/1,6-Mannosyltransferase	X		
AMDHD2	Amidohydrolase Domain Containing 2		X	
ANAPC11	Anaphase Promoting Complex Subunit 11			X
ASNA1	ArsA Arsenite Transporter, ATP-Binding, Homolog 1 (Bacterial)		X	
BSDC1	BSD Domain Containing 1		X	
CCDC189	Coiled-Coil Domain Containing 189	X	X	X
CEMP1	Cementum Protein 1		X	
CFHR3	Complement Factor H Related 3	X		
CIC	Capicua Transcriptional Repressor	X		
CTD-2144E22.5	Uncharacterized			X
EDF1	Endothelial Differentiation Related Factor 1	X		
ERF	ETS2 Repressor Factor	X		
ERLIN2	ER Lipid Raft Associated 2			X
FAM229A	Family With Sequence Similarity 229 Member A		X	
GSTK1	Glutathione S-Transferase Kappa 1			X
GSTT1	Glutathione S-Transferase Theta 1	X		
HDAC1	Histone Deacetylase 1		X	
KCTD3	Potassium Channel Tetramerization Domain Containing 3			X
KHDRBS1	KH RNA Binding Domain Containing, Signal Transduction Associated 1		X	
MAFG	MAF BZIP Transcription Factor G			X
MAL	Mal, T Cell Differentiation Protein	X		
MAMDC4	MAM Domain Containing 4	X		
MARCKSL1	MARCKS Like 1		X	
NLK	Nemo Like Kinase		X	X
NPB	Neuropeptide B			X
PCYT2	Phosphate Cytidylyltransferase 2, Ethanolamine			X
PDCD7	Programmed Cell Death 7	X	X	X
PHKG2	Phosphorylase Kinase Catalytic Subunit Gamma 2	X	X	X
PHPT1	Phosphohistidine Phosphatase 1	X		
PIGV	Phosphatidylinositol Glycan Anchor Biosynthesis Class V	X		
PLBD2	Phospholipase B Domain Containing 2	X		
PROSC	Pyridoxal Phosphate Binding Protein			X
PRSS27	Serine Protease 27		X	
RP11-863K10.7	Uncharacterized			X
SEC61B	Sec61 Translocon Beta Subunit	X		
SIRT7	Sirtuin 7			X
SPAG5	Sperm Associated Antigen 5		X	X
STARD7	StAR Related Lipid Transfer Domain Containing 7	X		
TMEM139	Transmembrane Protein 139	X	X	X
TRIR	Telomerase RNA Component Interacting RNase		X	
TSSK3	Testis Specific Serine Kinase 3		X	
ZNF703	Zinc Finger Protein 703	X		X

Table S9. Linear regression of total ROH length in megabases onto total local ancestry length in megabases by population.

	β_0 (p-value)	β_1 (p-value)	β_2 (p-value)
African American	$^{***}4.446 \times 10^{+2}$ ($< 2 \times 10^{-16}$)	$^{***}0.0784$ ($< 2 \times 10^{-16}$)	$^{***}0.0863$ ($< 2 \times 10^{-16}$)
Puerto Rican	$^{***}2.798 \times 10^{+2}$ ($< 2 \times 10^{-16}$)	$^{***}0.292$ ($< 2 \times 10^{-16}$)	$^{***}0.258$ ($< 2 \times 10^{-16}$)
Mexican American	-37.276 (0.291)	$^{***}0.3670$ ($< 2 \times 10^{-16}$)	$^{***}0.491$ ($< 2 \times 10^{-16}$)

β_0 is the regression coefficient for the intercept. β_1 is the regression coefficient for European Ancestry. β_2 is the regression coefficient for Native American ancestry. * p < 0.05, ** p < 0.01, *** p < 0.001.

Table S10. Gene regions with runs of homozygosity significantly enriched for European ancestry in Puerto Rican individuals.

See file TableS10.xlsx.

Table S11. Gene regions with runs of homozygosity significantly enriched for African ancestry in Puerto Rican American individuals.

chr:pos	Gene Symbol	p-value
chr6:30103885-30116512	*TRIM40*	8.57E-11
chr6:32361740-32374905	*BTNL2*	2.22E-10
chr6:29555683-29556745	*OR2H2*	6.79E-10
chr6:29364416-29365448	*OR12D2*	1.32E-09
chr6:28048753-28057341	*ZNF165*	2.71E-08
chr6:31514647-31526606	*NFKBIL1*	3.65E-07
chr6:31548302-31550299	*LTB*	7.20E-07
chr6:29523406-29601753	*GABBR1*	1.06E-06
chr6:31543344-31546113	*TNF*	1.13E-06
chr6:31539831-31542101	*LTA*	1.55E-06

Table S12. Gene regions with runs of homozygosity significantly enriched for Native American ancestry in Puerto Rican American individuals.

chr:pos	Gene Symbol	p-value
chr14:57671888-57673291	*AL391152.1*	4.90E-08
chr14:57735627-57756797	*AP5M1*	9.43E-08
chr14:57670518-57735726	*EXOC5*	1.45E-07
chr22:39348746-39359188	*APOBEC3A*	7.85E-07
chr2:54480315-54483409	*TSPYL6*	9.71E-07
chr14:57857262-57882635	*NAA30*	1.01E-06

chr9:140098534-140100090	*TMEM203*	2.44E-06

Table S13. Gene regions with runs of homozygosity significantly enriched for European ancestry in Mexican American individuals.

chr:pos	name	p-value
chr2:111271389-111334762	*RGPD6*	2.22E-12
chr15:48431625-48470714	*MYEF2*	1.71E-09
chr15:48413169-48434869	*SLC24A5*	1.78E-09
chr15:28623767-28637170	*GOLGA8F*	1.54E-07
chr15:42450899-42500514	*VPS39*	2.32E-07
chr1:248651885-248652898	*OR2T5*	4.18E-07
chr15:69745123-69748255	*RPLP1*	1.39E-06
chr17:34639793-34641846	*CCL4L2*	2.07E-06

Table S14. Gene regions with runs of homozygosity significantly enriched for African ancestry in Mexican American individuals.

chr:pos	Gene Symbol	p-value
chr6:30103885-30116512	*TRIM40*	2.50E-18
chr6:30585486-30594172	*MRPS18B*	6.25E-13
chr6:32361740-32374905	*BTNL2*	2.84E-12
chr6:30509154-30524951	*GNL1*	4.02E-12
chr6:30539153-30564956	*ABCF1*	1.60E-11
chr6:27106073-27114619	*HIST1H2BK*	1.84E-11
chr6:27107076-27108418	*HIST1H4I*	2.32E-11
chr6:30568177-30586389	*PPP1R10*	2.56E-11
chr6:26538633-26546482	*HMGN4*	3.92E-11
chr6:30524663-30531500	*PRR3*	1.58E-10
chr6:27093676-27100541	*HIST1H2BJ*	1.59E-10
chr6:30594619-30614600	*ATAT1*	1.75E-10
chr6:27100832-27103070	*HIST1H2AG*	1.94E-10
chr6:30667584-30685666	*MDC1*	2.76E-10
chr6:31865562-31913449	*C2*	3.37E-10
chr6:30152232-30181204	*TRIM26*	5.62E-10
chr6:30119722-30128711	*TRIM10*	8.15E-10
chr6:30644166-30655672	*PPP1R18*	1.42E-09
chr6:30614816-30620987	*C6orf136*	2.46E-09
chr6:30130993-30140473	*TRIM15*	2.57E-09
chr6:29555683-29556745	*OR2H2*	3.19E-09
chr6:27342394-27371683	*ZNF391*	3.34E-09
chr6:29364416-29365448	*OR12D2*	7.44E-09
chr6:29393281-29424848	*OR11A1*	1.04E-08
chr6:26501449-26510650	*BTN1A1*	1.09E-08

chr6:30655824-30659197	*NRM*	1.45E-08
chr6:29523406-29601753	*GABBR1*	1.96E-08
chr6:30457244-30461982	*HLA-E*	3.16E-08
chr6:27114861-27115317	*HIST1H2AH*	3.65E-08
chr6:31895475-31919825	*CFB*	4.71E-08
chr6:31895475-31919861	*CFB*	4.74E-08
chr6:31783291-31785723	*HSPA1A*	5.47E-08
chr6:30620896-30640814	*DHX16*	6.55E-08
chr6:31777396-31783437	*HSPA1L*	7.16E-08
chr6:32407619-32412823	*HLA-DRA*	7.38E-08
chr6:29407083-29408731	*OR10C1*	1.39E-07
chr6:26597180-26600278	*ABT1*	2.03E-07
chr6:26281283-26285762	*HIST1H4H*	2.14E-07
chr6:26225383-26225844	*HIST1H3E*	2.30E-07
chr6:31919864-31926887	*NELFE*	2.47E-07
chr6:26183958-26184454	*HIST1H2BE*	3.12E-07
chr6:26158349-26171577	*HIST1H2BD*	4.06E-07
chr6:26188938-26189304	*HIST1H4D*	4.10E-07
chr6:27418522-27440897	*ZNF184*	4.15E-07
chr6:26234440-26235216	*HIST1H1D*	5.55E-07
chr6:31795512-31798031	*HSPA1B*	5.59E-07
chr6:26217165-26217711	*HIST1H2AE*	6.30E-07
chr6:29323007-29399744	*OR5V1*	7.03E-07
chr6:26216428-26216872	*HIST1H2BG*	8.07E-07
chr6:26273144-26273622	*HIST1H2BI*	1.03E-06
chr6:29640169-29648887	*ZFP57*	1.39E-06
chr6:26240561-26240976	*HIST1H4F*	1.59E-06
chr6:26271146-26271612	*HIST1H3G*	1.71E-06
chr6:32485120-32498064	*HLA-DRB5*	2.47E-06

Table S15. Gene regions with runs of homozygosity significantly enriched for Native American ancestry in Mexican American individuals.

See file TableS15.xlsx.

Table S16. Gene regions with runs of homozygosity significantly enriched for European ancestry in African American individuals.

chr:pos	Gene Symbol	p-value
chr1:248651885-248652898	*OR2T5*	3.80E-10
chr1:248636627-248637634	*OR2T3*	3.24E-09
chr1:248721784-248722797	*OR2T29*	8.18E-09
chr1:248813232-248814185	*OR2T27*	3.33E-08
chr9:125512019-125513062	*OR1L6*	5.28E-08
chr20:39314488-39317880	*MAFB*	1.26E-07
chr13:64320934-64321323	*AL445989.1*	1.75E-07
chr6:3224495-3231964	*TUBB2B*	1.76E-07
chr20:45947246-45949467	*AL031666.2*	1.85E-07
chr1:68511645-68517314	*DIRAS3*	2.46E-07
chr1:248684916-248685964	*OR2G6*	3.07E-07
chr21:46031996-46032871	*KRTAP10-8*	3.10E-07
chr1:248616077-248617130	*OR2T2*	3.86E-07
chr17:61678231-61685725	*TACO1*	5.14E-07
chr9:125551150-125552237	*OR5C1*	1.05E-06
chr7:27282164-27290112	*EVX1*	1.06E-06
chr10:695888-711109	*PRR26*	1.10E-06
chr1:36023074-36032875	*NCDN*	1.18E-06
chr9:125437315-125438432	*OR1L3*	1.23E-06
chr14:105952654-105955284	*CRIP1*	1.37E-06
chr17:18427880-18430160	*FAM106A*	1.61E-06
chr1:248801588-248802559	*OR2T35*	1.68E-06
chr1:248185250-248186188	*OR2L5*	1.69E-06
chr3:128779610-128781249	*GP9*	1.74E-06
chr1:248201474-248202607	*OR2L2*	2.07E-06
chr4:71554196-71556267	*UTP3*	2.52E-06

Table S17. Gene regions with runs of homozygosity significantly enriched for African ancestry in African American individuals.

chr:pos	Gene Symbol	p-value
chr3:87276421-87304698	*CHMP2B*	4.12E-08
chr12:123745528-123756881	*CDK2AP1*	2.72E-07

Supplemental Methods

Processing of 1000 genomes data. Pemberton and Szpiech [1] published an analysis of deleterious variation and ROH in over 2,500 individuals from the 1000 Genomes Project [2] spanning the world. Included among these were individuals from six different population groups known to have non-trivial amounts of admixture ($n = 503$). In order to replicate our results, we obtained all the data from Pemberton and Szpiech [1] and extracted only the individuals from each of these six admixed populations populations: African American (ASW, n = 61), Afro-Caribbean (ACB, n = 95), Columbian (CLM, n = 94), Puerto Rican (PUR, n = 104), Peruvian (PEL, n = 85), and Mexican American (MXL, n = 64).

In order to call local ancestry tracts, we generated three putative ancestral populations using data from two sources. First, we obtained Affymetrix 6.0 genotyping data for both European (CEU, n = 99) and Yoruban (YRI, n = 108) individuals available from the 1000 Genomes Project [2]. Next, we obtained genotype data (n = 350) from the Native Mexican Diversity Panel (NMDP) [3], which were also typed on Affymetrix 6.0. The NMDP samples may have non-native admixture in them, and therefore we need to identify and exclude those with non-trivial amounts of inferred non-native ancestry from our reference panel. After merging these three data sets, we phase with SHAPEIT2 [4], and then we do an ADMIXTURE [5] analysis for k = 3 components and n = 20 replications, under the assumption that two of these components would identify European and African ancestry and the remaining component would be Native American. From the full set of data, we then filter anyone with < 99.9% ancestry from any given component. This leaves us with 86 CEU, 106 YRI, and 111 NMDP individuals, which we used as reference populations for RFMix [6] local ancestry inference of our 503 admixed samples.

ROH calls for each of these individuals were also extracted from the full set of data accompanying Pemberton and Szpiech [1], which were originally generated by Blant, et al. [7].

Deleteriousness information and counts were also obtained from Pemberton and Szpiech [1]. In their analysis, they used CADD scores [8]. CADD scores assign a numerical value to a mutation, with higher number suggesting more deleteriousness. For our analyses here, we combined all mutations with CADD $>= 15$ into the "damaging" category and all mutations with CADD $<= 15$ into the "benign" category.

We then reran the analysis described in the main text, finding qualitatively similar results (Figure S4).

1. Pemberton, T.J., and Szpiech, Z.A. (2018). Relationship between Deleterious Variation, Genomic Autozygosity, and Disease Risk: Insights from The 1000 Genomes Project. Am J Hum Genet 102, 658-675.
2. Genomes Project, C., Auton, A., Brooks, L.D., Durbin, R.M., Garrison, E.P., Kang, H.M., Korbel, J.O., Marchini, J.L., McCarthy, S., McVean, G.A., et al. (2015). A global reference for human genetic variation. Nature 526, 68-74.
3. Moreno-Estrada, A., Gignoux, C.R., Fernandez-Lopez, J.C., Zakharia, F., Sikora, M., Contreras, A.V., Acuna-Alonzo, V., Sandoval, K., Eng, C., Romero-Hidalgo, S., et al. (2014). Human genetics. The genetics of Mexico recapitulates Native American substructure and affects biomedical traits. Science 344, 1280-1285.
4. Delaneau, O., Zagury, J.F., and Marchini, J. (2013). Improved whole-chromosome phasing for disease and population genetic studies. Nat Methods 10, 5-6.
5. Alexander, D.H., Novembre, J., and Lange, K. (2009). Fast model-based estimation of ancestry in unrelated individuals. Genome Res 19, 1655-1664.

6. Maples, B.K., Gravel, S., Kenny, E.E., and Bustamante, C.D. (2013). RFMix: a discriminative modeling approach for rapid and robust local-ancestry inference. Am J Hum Genet 93, 278-288.
7. Blant, A., Kwong, M., Szpiech, Z.A., and Pemberton, T.J. (2017). Weighted likelihood inference of genomic autozygosity patterns in dense genotype data. BMC Genomics 18, 928.
8. Kircher, M., Witten, D.M., Jain, P., O'Roak, B.J., Cooper, G.M., and Shendure, J. (2014). A general framework for estimating the relative pathogenicity of human genetic variants. Nat Genet 46, 310-315.

Supplemental Acknowledgements

The “NHLBI TOPMed: Genes-environments and Admixture in Latino Asthmatics (GALA II) Study” was supported by NIH and NHLBI grant R01HL117004; study enrollment supported by NIEHS grant R01ES015794, the Sandler Family Foundation, the American Asthma Foundation, the RWJF Amos Medical Faculty Development Program, Harry Wm. and Diana V. Hind Distinguished Professor in Pharmaceutical Sciences II. All study collaborators: Shannon Thyne, UCSF; Harold J. Farber, Texas Children's Hospital; Denise Serebrisky, Jacobi Medical Center; Rajesh Kumar, Lurie Children's Hospital of Chicago; Emerita Brigino-Buenaventura, Kaiser Permanente; Michael A. LeNoir, Bay Area Pediatrics; Kelley Meade, Children's Hospital, Oakland; William Rodriguez-Cintron, VA Hospital, Puerto Rico; Pedro C. Avila, Northwestern University, Jose R. Rodriguez-Santana, Centro de Neumologia Pediatrica. The authors acknowledge the families and patients for their participation and thank the numerous health care providers and community clinics for their support and participation in GALA II. In particular, the authors thank study coordinator Sandra Salazar; the recruiters who obtained the data: Duanny Alva, MD, Gaby Ayala-Rodriguez, Lisa Caine, Elizabeth Castellanos, Jaime Colon, Denise DeJesus, Blanca Lopez, Brenda Lopez, MD, Louis Martos, Vivian Medina, Juana Olivo, Mario Peralta, Esther Pomares, MD, Jihan Quraishi, Johanna Rodriguez, Shahdad Saeedi, Dean Soto, Ana Taveras. See publication: PMID: 23750510.

The “NHLBI TOPMed: Study of African Americans, Asthma, Genes and Environment (SAGE)” was supported by NIH and NHLBI grants R01HL117004 and X01HL134589; study enrollment supported by the Sandler Family Foundation, the American Asthma Foundation, the RWJF Amos Medical Faculty Development Program, Harry Wm. and Diana V. Hind Distinguished Professor in Pharmaceutical Sciences II.
