# Supplementary material for: Ancestry-Dependent Enrichment of Deleterious Homozygotes in Runs of Homozygosity
Source: Am J Hum Genet. 2019 Sep 19;105(4):747–62. doi: 10.1016/j.ajhg.2019.08.011 (PMC6817522; doi:10.1016/j.ajhg.2019.08.011)
Supplement: Document S2. Article plus Supplemental Data [file mmc4.pdf]

Ancestry-Dependent Enrichment of Deleterious Homozygotes in Runs of Homozygosity

Zachary A. Szpiech,[1,2,*] Angel C.Y. Mak,[3] Marquitta J. White,[3] Donglei Hu,[3] Celeste Eng,[3] Esteban G. Burchard,[3] and Ryan D. Hernandez[1,4,5,6,7,*]

Runs of homozygosity (ROH) are important genomic features that manifest when an individual inherits two haplotypes that are identical by descent. Their length distributions are informative about population history, and their genomic locations are useful for mapping recessive loci contributing to both Mendelian and complex disease risk. We have previously shown that ROH, and especially long ROH that are likely the result of recent parental relatedness, are enriched for homozygous deleterious coding variation in a worldwide sample of outbred individuals. However, the distribution of ROH in admixed populations and their relationship to deleterious homozygous genotypes is understudied. Here we analyze whole-genome sequencing data from 1,441 unrelated individuals from self-identified African American, Puerto Rican, and Mexican American populations. These populations are three-way admixed between European, African, and Native American ancestries and provide an opportunity to study the distribution of deleterious alleles partitioned by local ancestry and ROH. We re-capitulate previous findings that long ROH are enriched for deleterious variation genome-wide. We then partition by local ancestry and show that deleterious homozygotes arise at a higher rate when ROH overlap African ancestry segments than when they overlap European or Native American ancestry segments of the genome. These results suggest that, while ROH on any haplotype background are associated with an inflation of deleterious homozygous variation, African haplotype backgrounds may play a particularly important role in the genetic architecture of complex diseases for admixed individuals, highlighting the need for further study of these populations.

Introduction

Runs of homozygosity (ROH) are long stretches of identical-by-descent (IBD) haplotypes that manifest in individual genomes as the result of recent parental relatedness. Originally conceived to improve the accuracy of homozygosity mapping of recessive Mendelian diseases, ROH have formed the foundation of studies investigating the contribution of recessive deleterious variants to the genetic risk for complex diseases and to the determination of complex traits.[1] Moreover, they have provided unique insights into the demographic and sociocultural processes[1] that have shaped genomic variation patterns in contemporary worldwide human populations,[2–8] ancient hominins,[9–12] non-human primates,[13,14] woolly mammoths,[15] livestock,[16–21] birds,[22,23] felines,[24] and canids.[25–31] Recent population bottlenecks, cultural preferences for endogamy or consanguineous marriage, and natural selection can create increased rates of ROH in individual genomes, substantially increasing overall homozygosity in such populations.

Several studies of the distribution of ROH in ostensibly outbred human populations have shown that ROH are common and range in size from tens of kilobases to several megabases in length.[2–5] Furthermore, total length and prevalence of ROH are correlated with distance from Africa,[3–5] with more and longer ROH manifesting in individuals from populations a longer distance away. These patterns likely reflect increased IBD among haplotypes as a result of the serial bottlenecking process that humans experienced as they migrated out of Africa.

The prevalence of ROH in individual genomes has also been an important factor for understanding the genetic basis of complex phenotypes.[32–34] High levels of ROH have been associated with heart disease,[35,36] cancer,[37–39] blood pressure,[40,41] LDL cholesterol,[41] various mental disorders,[42–45] human height,[46,47] and increased susceptibility to infectious diseases.[48] Indeed, these results are consistent with the idea that many rare alleles of small effect may be the cause of increased risk for complex diseases,[49–51] especially if these mutations are recessive.[2]

We have previously shown that ROH, especially long ROH, are enriched for deleterious homozygous variation.[52,53] Whereas an overall increase in homozygotes is expected with increasing genomic ROH, we have shown that the rate at which deleterious homozygotes accumulate outpaces the rate at which benign homozygotes accumulate[52,53] in long ROH (ROH on the order of several megabases). This is a consequence of young (long) haplotypes containing low-frequency variants getting paired IBD.[53] As low-frequency variants are more likely to be deleterious than common variants, the processes that create very

[1]Department of Bioengineering and Therapeutic Sciences, University of California San Francisco, San Francisco, CA 94158, USA; [2]Department of Biological Sciences, Auburn University, Auburn, AL 36842, USA; [3]Department of Medicine, University of California San Francisco, San Francisco, CA 94158, USA; [4]Institute for Human Genetics, University of California San Francisco, San Francisco, CA 94158, USA; [5]Quantitative Biosciences Institute, University of California San Francisco, San Francisco, CA 94158, USA; [6]Department of Human Genetics, McGill University, Montreal, QC H3A 0G1, Canada; [7]Genome Quebec Innovation Center, McGill University, Montreal, QC H3A 0G1, Canada
*Correspondence: zas0020@auburn.edu (Z.A.S.), ryan.hernandez@me.com (R.D.H.)
https://doi.org/10.1016/j.ajhg.2019.08.011.
© 2019 The Authors. This is an open access article under the CC BY license (http://creativecommons.org/licenses/by/4.0/).

long ROH can also generate unusually high numbers of deleterious homozygotes within these regions.

Although a few studies describing the worldwide distribution of ROH patterns have included a small number of admixed populations,[3–5] the number of individuals per admixed population has been fairly small. Even as the number of admixed individuals continues to grow in the United States,[54] they are still relatively understudied, which translates to disparities in our understanding of population-specific genetic factors that may influence complex phenotypes.[55] Indeed, admixed populations have unique features compared to other populations, in that genomes from these populations are recent combinations of two or more ancestral populations.

This ancestral mosaicism has been exploited to make inferences about the natural history of human populations[56–63] and to search for ancestral haplotypes that influence complex phenotypes.[64–68] Here we add to the body of work on admixed populations by examining the relationship between ROH, local ancestry, and the accumulation of deleterious alleles. We use 1,441 recently published[69] whole-genome sequences distributed roughly equally across three admixed populations in the Americas: African American (n = 475), Mexican American (n = 483), and Puerto Rican (n = 483). Each of these populations is three-way admixed, with distinct contributions from European, Native American, and African ancestral populations.

Among the ancestral populations that contributed haplotypes to these admixed populations, it has been shown that the distribution of deleterious heterozygotes and deleterious homozygotes changes with distance from Africa.[70–73] With this in mind, we propose that accumulation of deleterious homozygotes via increased genomic ROH may also differ within admixed populations based on differing ancestral haplotypes. Indeed, with high deleterious heterozygosity, we propose that African ancestral haplotypes may be most susceptible to large increases in deleterious homozygotes when subjected to harsh bottlenecks or inbreeding, as these low-frequency deleterious alleles will be paired into homozygotes as a result of increased genomic ROH.

Material and Methods

Sample Selection and Quality Control

We used 1,441 whole-genome sequences (dbGaP accession numbers phs000920 and phs000921) from three different admixed populations: African American (n = 475), Mexican American (n = 483), and Puerto Rican (n = 483). These data are an unrelated (up to third-degree relative) set that were previously published by Mak et al.,[69] who previously identified and removed third-degree (and closer) relatives and conducted all QC. These genomes all had mean genome coverage >30× with >95% of genome covered at >10× and were called with GATK HaplotypeCaller. Site-level QC was conducted via GATK Variant Quality Score Recalibration, filtering at the 99.8% tranche. Individual genotypes were filtered if they did not have a minimum read depth of 10 and genotype quality of 20. Full details are available in Mak et al.[69]

Calling Local Ancestry

We used 90 African (YRI) individuals and 90 European (CEU) individuals for ancestry references (genotypes obtained from the Axiom Genotype Dataset, see Web Resources) and SNPs with less than 95% call rate were removed. For Native American reference genotypes, we used 71 Native American individuals previously genotyped on the Axiom Genome-Wide LAT 1 array.[74] These samples are unrelated and unadmixed individuals including 14 Zapotec, 2 Mixe, and 11 Mixtec from the southern Mexican state of Oaxaca[75] and 44 Nahua individuals from Central Mexico.[76] Although these individuals are unlikely to exactly match the Native components of all the individuals in our sample, they act as a reasonable proxy for inferring those components, just as our YRI and CEU reference populations act as a reasonable proxy for inferring the African and European components, respectively.

We then subset our 1,441 whole-genome sequences corresponding to sites found on the Axiom Genome-Wide LAT 1 array, leaving 765,321 markers. We then merge these data with our European (CEU), African (YRI), and Native American (NAM) reference panels, which overlapped at 434,145 markers. After filtering multi-allelic SNPs and SNPs with >10% missing data, we obtained a final merged dataset of 428,644 markers. We phased this combined dataset using SHAPEIT2[77] and called local ancestry tracts jointly with RFMix[78] under a three-way admixture model based on the African, European, and Native American reference genotypes described above.

Calling Runs of Homozygosity

We called runs of homozygosity using the program GARLIC v.1.1.4,[79] which implements the ROH calling pipeline of Pemberton et al.[4] for each population separately on the full whole-genome call set, filtering only monomorphic sites. For the 475 African American (AA) individuals, this left 39,517,679 segregating sites; for the 483 Puerto Rican (PR) individuals, this left 31,961,900 segregating sites; and for the 483 Mexican American (MX) individuals, this left 30,744,389 segregating sites. Instead of asserting a single constant genotyping error rate (as in Pemberton et al.[4]), we used genotype quality scores provided with the WGS data to give GARLIC a per-genotype estimation of error. Using GARLIC's rule of thumb parameter estimation, we chose analysis window sizes of 290 SNPs, 250 SNPs, and 210 SNPs and overlap fractions of 0.3688, 0.3553, and 0.3528 for the AA, PR, and MX populations, respectively. GARLIC chose LOD score cutoffs of −47.5169, −70.1977, and −60.9221 for the AA, PR, and MX populations, respectively. Using a three-component Gaussian mixture model, GARLIC determined three size classes: small class A, medium class B, and long class C ROH. Class A/B and class B/C size boundaries were inferred as 38,389 bps and 142,925 bps for AA; as 50,618 bps and 230,079 bps for PR; and 46,979 bps and 217,054 bps for MX.

Computing Ancestry Enrichment in ROH

To determine whether the ROH covering a gene region is overrepresented for a particular ancestry, we first compute, for each gene region, the quantities A_i^R and N^R, which represent the mean proportion of ancestry i in ROH at gene region R and the "number" of ROH in each gene region, respectively. Note that if an ROH

only covers part of a gene region, then only that fraction is counted, thus N^R is continuous and not a whole number. We also compute the mean proportion of ancestry i in the population, A_i. If we consider the fraction of ancestry type i in ROH (A_i^R) as a random sample from the distribution of ancestry in the population (A_i), then we can model the ancestry-specific ROH sampling process with a beta distribution. This is conceptually similar to a binomial sampling process, where sampling ancestry i in an ROH is considered a "success" but in continuous space. Here we wish to compute the probability of sampling $\mathrm{N}^R A_i^R$ ROH regions of ancestry i (or more) given that the population admixture fraction of ancestry i is A_i and that we have N^R ROH total. We can do this by computing $P[x \geq N^R A_i^R \mid N^R, A_i] = I_{A_i}(\mathrm{N}^R A_i^R + 1, N^R - \mathrm{N}^R A_i^R)$, where $I_p(\mathrm{a}, b)$ is the regularized incomplete beta function.

Calling Deleterious Alleles

Using the Whole Genome Sequencing Annotation (WGSA) pipeline[80] to generate annotation data, we extracted PolyPhen 2,[81] SIFT,[82] Provean,[83] and GERP[84] scores for deleteriousness, as well as high-confidence ancestral allele states (from Enredo-Pecan-Ortheus alignments) and synonymous annotations and for all mutations in coding regions (WGSA pre-computed annotations available online, see Web Resources).

PolyPhen 2 generates three deleteriousness categories: Probably Damaging, Possibly Damaging, and Benign. If a mutation has more than one PolyPhen2 classification (e.g., Benign and Probably Damaging), it is reassigned to have only the most damaging category of the group. All mutations that have a PolyPhen 2 prediction or that are synonymous are then pooled into two separate categories: "damaging" and "benign." All Probably Damaging or Possibly Damaging mutations are pooled into the "damaging" category, and all Benign and synonymous mutations are pooled into the "benign" category.

SIFT generates two deleteriousness categories, Intolerant and Tolerant, which we relabel "damaging" and "benign." If a mutation has more than one SIFT classification, it is reassigned to have only the most damaging category of the group.

Provean generates two deleteriousness categories, Deleterious and Neutral, which we relabel "damaging" and "benign." If a mutation has more than one Provean classification, it is reassigned to have only the most damaging category of the group.

GERP generates a numerical score at a given locus where a higher score indicates more deleteriousness for a derived allele at that locus. Here we focus on derived alleles that are very likely to be deleterious and combine all derived mutations at sites with GERP $\geq$ 6 into the category "damaging." We form our "benign" category with all derived mutations with GERP $\leq$ 2.

Defining Gene Sets

We sought to define three sets of genes for further analysis based on the probability of intolerance to loss of function (pLI) predicted as part of the gnomAD project[85] (Web Resources). This score ranges from 0 to 1, with high scores suggesting an intolerance to inactivation and low scores suggesting a tolerance for inactivation. The distribution of these scores is bimodal, with most genes having a pLI near 0 or 1. Of the 18,451 autosomal genes with a pLI score, we create a "low-pLI" category consisting of 13,128 genes with a $pLI \leq 0.2$ and a "high-pLI" category consisting of 3,241 genes with a $pLI \geq 0.8$. We finally create an "all" category consisting of all 18,451 autosomal genes reported as part of the gnomAD project.[85]

Computing Minor Allele Frequencies

In order to determine minor allele frequency (MAF) category, we use frequencies computed from all TOPMed[86] Freeze 3 whole-genome sequencing datasets (dbGaP accession numbers phs000920, phs000921, phs001062, phs001032, phs000997, phs000993, phs001189, phs001211, phs001040, phs001024, phs000974, phs000956, phs000951, phs000946, phs000988, phs000964, phs000972, phs000954, and phs001143) forming a total sample size of n = 18,581. We then categorize variants in the dataset analyzed here as common (MAF $\geq$ 0.05) and rare (MAF < 0.05) based on these "global" allele frequencies.

Simulations

We perform simulations to examine how demographic history affects the concentration of deleterious homozygotes in ROH. We use the forward simulation program SLiM 3[87,88] to simulate deleterious mutations within a complex demography in conjunction with the coalescent simulator msprime[89] to simulate neutral mutations conditional on the forward simulation genealogy. This allows us to efficiently simulate very large genomic regions, which is a requirement for analyzing the distribution of long ROH that typically extend several megabases. We complete 500 replicates of the following simulations.

We simulate a three-population demographic history after Gravel et al.[90] in SLiM 3, introducing recessive mildly deleterious alleles with selection coefficients drawn from $\Gamma(-0.03, 0.2)$. We simulate a 100 Mbps region, where deleterious alleles are allowed to occur in designated "coding regions." These regions are defined based on the hg19 exon coordinates of all CCDS genes in the first 100 Mbps of human chromosome 1. Similarly, we simulate a variable recombination rate based on the HapMap phase II[91] inferred map. We allow a mutation rate based on the Gravel et al.[90] inferred mutation rate of 2.36×10^{-8}, setting the deleterious mutation rate at one-tenth of this value. At the end of the forward simulation, a list of segregating deleterious mutations and their genomic locations is output along with the full tree sequence[88,89] of the entire simulation. Neutral mutations are then added with msprime.[89]

To simulate neutral mutations conditional on the forward simulation history, we load our population tree sequence with the pyslim package,[88] recapitate to ensure all lineages fully coalesce, and then lay down neutral mutations at a rate of 90% of the Gravel-inferred rate (so that the neutral plus deleterious mutation rate equals the inferred rate). Finally, we sample 500 diploid individuals from each population in the simulation for analysis.

Simulation code is available online (see Web Resources).

Results

Admixture

Using the subset of sites from our whole-genome sequencing data that intersected with our African, European, and Native American reference panels, we called 3-way local ancestry tracts in all 1,441 samples (see Material and Methods). We also estimated global ancestry proportions by summing the length of all haplotypes inferred to be from a given ancestry and dividing by the total genome length. Figure 1 summarizes the global ancestry proportions for all individuals from each population on a ternary plot. The admixture proportions largely accord with previous results in these populations, with Puerto

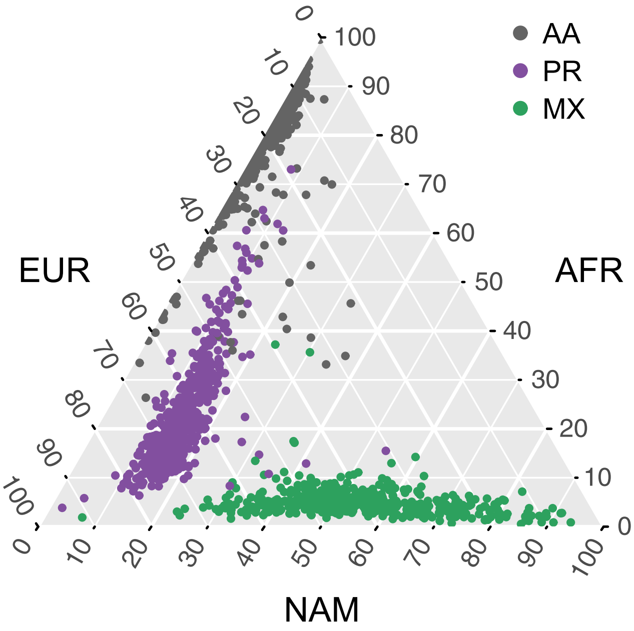

Figure 1. A Ternary Plot of Global Ancestry Proportions
Each point represents a single individual, with their global ancestry proportions shown on each of the three axes (European, EUR; African, AFR; and Native American, NAM). Individuals are colored based on their reported ethnicity, with African Americans (AA) colored gray, Puerto Ricans (PR) colored purple, and Mexican Americans (MX) colored green.

Ricans having mostly African and European ancestry, Mexican Americans having mostly European and Native American ancestry, and African Americans having mostly African and European ancestry to the near exclusion of any Native American ancestry. However, although African Americans are frequently treated as a 2-way admixed population between European and African sources, we show that several AA individuals have non-trivial proportions of Native American ancestry. This suggests that, in general, a 2-way admixture model may not be uniformly appropriate for studying admixture patterns among self-identified African American individuals.

Runs of Homozygosity

We followed the ROH calling pipeline of Pemberton et al.[4] as implemented in the software GARLIC[79] to call ROH from the full whole-genome sequencing data (see Material and Methods). This method identifies three classes of ROH based on the length distribution in each population. We refer to these size classes as short, medium, and long. These classes roughly correspond to ROH formed of IBD haplotypes from different time periods from the population history. Short ROH are tens of kilobases in length and likely reflect the homozygosity of old haplotypes; medium ROH are hundreds of kilobases in length and likely reflect background relatedness in the population; and long ROH are hundreds of kilobases to several megabases in length and are likely the result of recent parental relatedness. Total length of ROH in the genome is correlated with distance from Africa.[2,4] In the case of our admixed populations, we therefore expect the total length of ROH to be correlated with increased European and Native American admixture fraction. Figure 2A illustrates this pattern, with AA individuals having lowest total ROH, PR individuals having intermediate total ROH, and MX individuals having the highest total ROH (all pairwise Mann-Whitney U tests $p < 2.2 \times 10^{-16}$). Indeed, if we do multiple regression of total ROH coverage (in Mbps) onto total European and total Native American coverage (in Mbps), we find a significant positive association with both ancestry backgrounds in all three populations (Table S9). Breaking down ROH by size class, we find that the total length of short ROH is similar but still significantly higher in PR than in MX individuals ($p < 2.2 \times 10^{-16}$; Figure 2B), but the total length of both medium ROH ($p < 2.2 \times 10^{-16}$; Figure 2C) and total long ROH ($p < 2.2 \times 10^{-16}$; Figure 2D) is highest on average in MX individuals.

As it has been previously noted that ROH do not occur uniformly across the genome,[4,5] we also examined the proportion of ROH coverage of each of 18,451 coding genes from the gnomAD project[85] across all individuals in each population to discover whether certain genes or sets of genes were enriched for ROH coverage. For each gene region (exons plus introns), we compute the fraction of basepairs that are covered by ROH in each individual and take the mean of this fraction across individuals. Next, we look at the top 0.1% of genes with the highest overall ROH coverage across individuals in each population (Table S8). This corresponds to genes with greater than 0.661, 0.891, and 0.971 ROH coverage across individuals in the African American, Puerto Rican, and Mexican populations, respectively. Although none of these gene sets were enriched for any gene ontology terms, four gene regions were found in all populations: CCDC189, PDCD7, PHKG2, and TMEM139.

We also examine whether certain gene sets may have more enrichment for ROH than others. In particular we create two gene sets based on the gnomAD project's[85] predicted intolerance to loss of function (pLI) measurement (see Material and Methods). The high-pLI gene set consists of 3,241 genes predicted to be most intolerant to loss of function in humans, and the low-pLI gene set consists of 13,128 genes predicted to be least intolerant to loss of function in humans.

Table 1 lists the means and ranges for ROH coverage across individuals for both high-pLI and low-pLI gene sets. Although the ranges tend to span most of the [0,1] interval, we do observe a small but significant difference in the mean ROH coverage between high-pLI and low-pLI gene sets (as tested by a two-sided Mann-Whitney U test) across all populations, with high-pLI genes having slightly more ROH on average. This may be a result of high-pLI genes experiencing stronger background selection, as high-pLI genes are intolerant to loss of function in humans and mutations in these genes may therefore be more deleterious on average. This, in turn, may contribute a

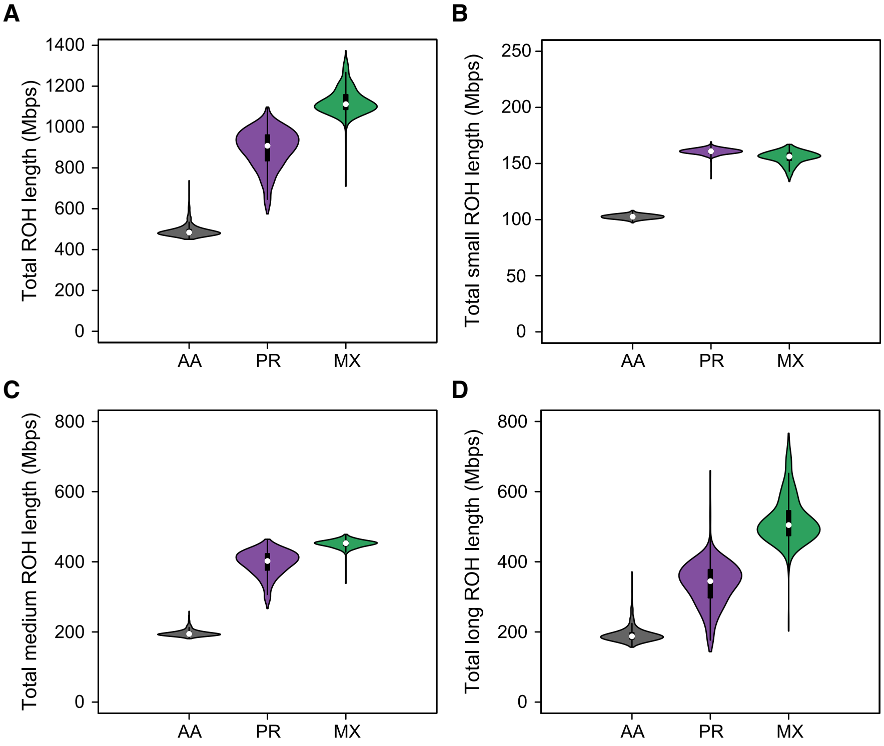

Figure 2. The Distribution of Summed ROH Lengths across Size Classes
Shown are (A) all ROH, (B) short ROH, (C) medium ROH, and (D) long ROH. AA, African American; PR, Puerto Rican; MX, Mexican American.

non-trivial amount of homozygosity to the patterns of ROH we observe.

We also tested whether ROH in certain gene regions are overrepresented with one ancestry background relative to the distribution of ancestries at that gene region population-wide. We compute the probability of observing as much or more of each ancestry among the set of ROH at a gene region for all populations (see Material and Methods) for each 18,451 gene regions from the gnomAD project. Significance was determined via Bonferroni correction, and we find numerous genes in each population enriched for various ancestries (Tables S10, S11–S14, S15, S16, and S17). Each population had at least one gene enriched for each ancestry, except African Americans, where we found no genes enriched for Native American ancestry (though the proportion of Native American ancestry in this population is low, ~2%, so power may be limited).

We conduct a gene ontology (GO) enrichment analysis using PantherDB,[92] as some of the enrichment lists were large. We find among genes enriched for African ancestry in Mexican Americans significant enrichment of GO terms related to nucleosome assembly (FDR $= 2.13 \times 10^{-5}$), cellular response to unfolded protein (FDR $= 6.95 \times 10^{-3}$), and cellular response to heat (FDR $= 1.22 \times 10^{-2}$). Among genes enriched for Native American ancestry in Mexican Americans we find significant enrichment of GO terms related to spindle assembly (FDR $= 1.27 \times 10^{-2}$) and detection of chemical stimulus involved in sensory perception (FDR $= 3.59 \times 10^{-3}$). Finally, we also find among genes enriched for African ancestry in Puerto Ricans significant enrichment of GO terms related to cytokine-mediated signaling pathways (FDR $= 2.27 \times 10^{-2}$).

Deleterious Alleles

We used multiple approaches to predict the deleteriousness of all sites in the genome (see Material and Methods), but focus on missense mutations classified as Probably Damaging, Possibly Damaging, or Benign using PolyPhen 2.[81] As in Szpiech et al.,[52] we combine the Probably Damaging and Possibly Damaging mutations into a single "damaging" class, and we combine all Benign mutations with synonymous mutations into a single "benign" class. For individual i across all sites, we denote by $g_i^{d,k}$ and $g_i^{b,k}$ the total number of sites with $k \in \{0, 1, 2\}$ alternate alleles classified as damaging or benign, respectively. In Figure 3A we plot the distribution of deleterious heterozygotes per individual, $g_i^{d,1}$, split by population. Consistent with previous work,[70–73] we see an increased number of deleterious heterozygotes in populations with more African ancestry, with AA individuals having the most and MX individuals having the fewest (patterns replicate with other deleterious categories, see Figures S5–S10). Conversely, we would expect an increase of deleterious homozygotes per individual in populations with more non-African ancestry. Indeed, in Figure 3B we plot the distribution of deleterious homozygotes per individual, $g_i^{d,2}$, split by population and observe AA individuals with the fewest and MX individuals having the most (these patterns also

Table 1. Range and Mean ROH Coverage of High-pLI and Low-pLI Gene Sets by Population

Population	High-pLI Genes Range	High-pLI Genes Mean	Low-pLI Genes Range	Low-pLI Genes Mean	Difference of Means (p value)
AA	$[0.013, 0.699]$	0.195	$[0, 0.818]$	0.181	***$< 2 \times 10^{-16}$
PR	$[0.023, 0.914]$	0.346	$[0, 0.974]$	0.329	***1.196×10^{-9}
MX	$[0.019, 0.977]$	0.428	$[0, 0.992]$	0.414	***1.586×10^{-5}

p value for difference of means computed by two-sided Mann-Whitney U test. *$p < 0.05$, **$p < 0.01$, ***$p < 0.001$.

replicate with other deleterious categories, see Figures S5–S10). Figure 3C plots the total number of deleterious alleles per individual ($g_i^{d,1} + 2g_i^{d,2}$). Contrary to other work,[73] we find a total deleterious load highest on average in AA individuals. Although this pattern replicates across several other deleterious calling methods (Figures S5–S9), when using GERP scores (as in Henn et al.[73]), the pattern reverses (Figure S10) and is consistent with Henn et al.[73]

Deleterious Alleles across Local Ancestry

We next investigate whether there are any differences in deleterious load by local ancestry. Although our local ancestry calls provide us with phased local ancestry inferences, we were limited to a small subset of sites for our reference populations. Since the vast majority of our deleterious alleles come from our unphased whole-genome data, we do not have phase information for the deleterious alleles and cannot assign a specific ancestral haplotype in regions of discordant ancestry. Therefore, we calculate total load based on six different ancestry backgrounds. AFR, EUR, and NAM ancestry regions represent regions that are homozygous for African, European, and Native American ancestries, respectively, and AFEU, EUNA, and AFNA ancestry regions represent regions that are called heterozygous for African/European, European/Native American, and African/Native American ancestries, respectively. We then calculate for each population the number of deleterious alleles per basepair for each ancestry background.

Table 2 shows the number of deleterious alleles per basepair for each population and each ancestry background using PolyPhen 2 deleterious calls (results were qualitatively similar across all other deleterious call sets). We perform two types of tests for independence in order to determine whether there are significant differences in the number of deleterious alleles per basepair. First, we test for independence of the count of deleterious alleles on an ancestry background and the count of basepairs covered by that ancestry across populations. We find that neither African ancestry nor European ancestry have statistical differences in the number of deleterious alleles per MB across populations. Further, while NAM, EUAF, and AFNA exhibit statistically differences across populations, it appears to be driven by one of the two populations (AA, MX, and PR, respectively). Next, we test for independence of these counts across ancestries within each population. Here we find that all populations have statistically significant differences in the distribution of deleterious alleles across ancestry backgrounds (AA $p < 2.2 \times 10^{-16}$; MX $p < 2.2 \times 10^{-16}$; PR $p < 2.2 \times 10^{-16}$), with NAM ancestry having the lowest rate in AA and PR individuals and EUR having the lowest rate in MX individuals. However, we note that the overall differences were very small (a difference of <0.1 deleterious alleles per Mbp).

Deleterious Alleles in ROH

Next, we turn to examining the distribution of deleterious homozygotes within ROH. It was previously reported[52,53] that there is a higher proportion of deleterious homozygotes per unit increase of ROH than expected from the proportion of benign homozygotes. Naturally, as the total amount of genomic ROH increases, we expect more homozygotes to fall within ROH. However, Szpiech et al.[52] and Pemberton and Szpiech[53] found that the rate of increase of the proportion of deleterious homozygotes was greater than for benign homozygotes. This effect was strongest for long ROH, which are likely the result of recent parental relatedness.

For each individual i and for each ROH class $j \in \{A, B, C, R, N\}$ (A, short ROH; B, medium ROH; C, long ROH; R, all ROH; and N, outside ROH), we define the number of damaging or benign sites with $k \in \{0, 1, 2\}$ alternate alleles as $g_{i,j}^{d,k}$ and $g_{i,j}^{b,k}$, respectively. Thus, we calculate the proportion of damaging homozygotes in ROH class j as

$$f_{i,j}^{d} = \frac{g_{i,j}^{d,2}}{g_{i,R}^{d,2} + g_{i,N}^{d,2}}$$

and the proportion of benign homozygotes in ROH as

$$f_{i,j}^{b} = \frac{g_{i,j}^{b,2}}{g_{i,R}^{b,2} + g_{i,N}^{b,2}},$$

respectively. We also compute, for each individual i and each class j, the fraction of the genome covered in ROH as

$$G_{i,j} = \frac{\textit{total length of ROH regions of class j in individual i}}{\textit{total genome length}}.$$

We plot the proportions of ROH homozygotes versus genomic fraction of ROH in Figure 4, which is analogous to Figure 4 from Szpiech et al.[52] In order to determine whether there is a statistically significant difference in the accumulation of deleterious homozygotes versus

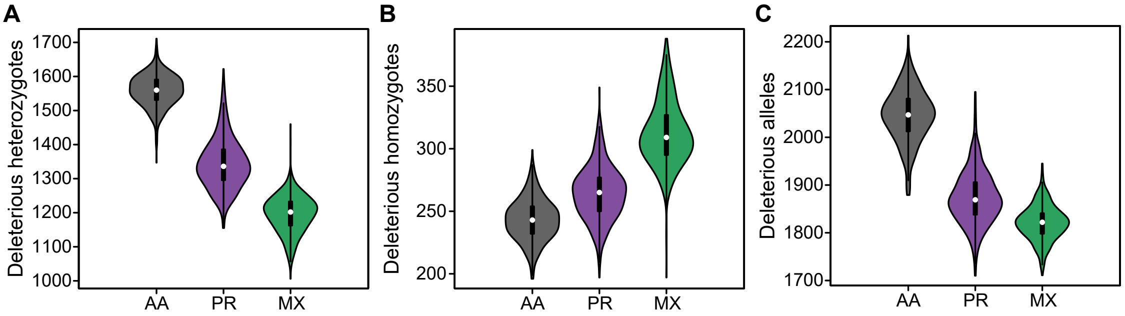

Figure 3. The Distribution of Deleterious Alleles across Populations
The number of (A) deleterious heterozygotes, (B) deleterious homozygotes, and (C) total deleterious alleles per individual using PolyPhen2 classifications. AA, African American; PR, Puerto Rican; MX, Mexican American.

benign homozygotes, we construct a linear regression model (as in Szpiech et al.[52] and Pemberton and Szpiech[53]), $f_{\cdot j} = \beta_0 + \beta_1 G_{\cdot j} + \beta_2 D + \beta_3 DG_{\cdot j} + \varepsilon$, where $f_{\cdot j}$ is a vector of length 2,882 containing the proportions of both damaging and benign homozygotes in ROH class j for all individuals, $G_{\cdot j}$ is a vector of genomic class j ROH proportions, and D is an indicator variable taking a value of 1 when the response represents damaging homozygotes and 0 for benign homozygotes. In this framework, a statistically significant β_2 suggests an overall higher proportion of damaging homozygotes in ROH compared to benign homozygotes, e.g., $\beta_2 = 0.1$ means that an extra 10% of genome-wide deleterious homozygotes fall in ROH compared to the distribution of benign homozygotes. A statistically significant β_3 suggests a difference in the rate of accumulation per unit increase of ROH, e.g., $\beta_3 = 1.0$ means that for a 10% increase in genomic ROH, 10% more deleterious homozygotes fall in ROH compared to benign homozygotes. Inferred coefficients for the four regressions corresponding to each $j \in \{A, B, C, R\}$ are given in Table S1.

Figure 4A plots these proportions versus total ROH for all ROH classes combined. In agreement with Szpiech et al.,[52] we find that there is an overall greater proportion of damaging homozygotes in ROH compared to benign homozygotes ($\beta_2 = 0.1799$, $p < 2 \times 10^{-16}$), but in contrast the overall rate of accumulation is not different ($\beta_3 = 1.807 \times 10^{-2}$, $p = 0.0671$). When we partition ROH by size class, the distribution of homozygotes in short ROH (Figure 4B) also differs from Szpiech et al.[52] Whereas previously there were no statistically significant differences in β_2 or β_3, here we find a significant positive $\beta_2 = 4.810 \times 10^{-2}$ ($p < 2 \times 10^{-16}$) and a statistically significant negative $\beta_3 = -0.428$ ($p < 1.10 \times 10^{-8}$), suggesting that ROH comprised of old haplotypes accumulate deleterious homozygotes at a slower rate that benign homozygotes. As we expect short ROH to be comprised of old haplotypes that have been segregating for a long time, it is reasonable to think that only haplotypes with relatively few deleterious alleles remain segregating in the population. Our results for medium (Figure 4C) and long ROH (Figure 4D) are consistent with previous work;[52,53] in particular we find that the difference in rates of gain of deleterious versus benign homozygotes is greatest in long ROH ($\beta_3 = 0.229$; $p < 2 \times 10^{-16}$).

We also consider whether we can detect a difference in concentration of deleterious homozygotes in our high-pLI and low-pLI gene sets. For this analysis we only consider predicted deleterious homozygotes, and we wish to compare the genome-wide proportion of these genotypes between high-pLI and low-pLI genes. To do this we construct the following linear regression, $f_{\cdot j} = \beta_0 + \beta_1 G_{\cdot j} + \beta_2 H + \beta_3 HG_{\cdot j} + \varepsilon$, where $f_{\cdot j}$ and $G_{\cdot j}$are as above and H is an indicator variable taking a value of 1 or 0 if the response comes from the high-pLI gene set or the low-pLI gene set, respectively (Table S4). Here β_3 represents the difference in rate of accumulation of deleterious homozygotes in high-pLI genes versus low-pLI genes. We find a significant difference in the accumulation of deleterious homozygotes in high-pLI genes versus low-pLI genes for total ROH ($\beta_3 = 7.243 \times 10^{-2}$, $p = 0.0253$) and short ROH ($\beta_3 = 0.502$, $p = 0.0359$), although not for long ROH ($\beta_3 = 9.639 \times 10^{-2}$, $p = 0.0960$) or medium ($\beta_3 = -2.262 \times 10^{-2}$, $p = 0.774$). In this analysis we compare damaging alleles across two gene sets (instead of comparing damaging to non-damaging), where we might expect mutations in loss-of-function intolerant genes (high-pLI) to be more deleterious compared to mutations in loss-of-function tolerant genes (low-pLI). In this case, the effect size may be much smaller, and by restricting our high-pLI gene set to such a small number of genes we may lack power to detect it. However, in aggregate these results suggest that a higher proportion of genome-wide deleterious homozygotes fall within high-pLI genes versus low-pLI genes.

Deleterious Alleles in ROH Partitioned by Local Ancestry

Now we turn to analyzing the distribution of deleterious homozygotes in ROH comprised of only one particular ancestral haplotype. As shown in Figure 3A and in other work,[70–73] populations with more African ancestry tend to have high numbers of deleterious heterozygotes genome-wide. This contrasts with populations that have

Table 2. The Number of Deleterious Alleles per Megabase Partitioned by Population and Local Ancestry Background

	AFR (p=0.160)	EUR (p=0.452)	NAM*** ($p=3.314\times10^{-7}$)	EUAF** ($p1.131\times10^{-3}$)	EUNA (p=0.123)	AFNA** ($p=4.392\times10^{-3}$)
AA*** ($p < 2 \times 10^{-16}$)	0.335 (1.642×10^{6})	0.284 (1.009×10^{5})	0.237 (8.648×10^{2})	0.311 (7.943×10^{5})	0.280 (2.491×10^{4})	0.315 (8.364×10^{4})
PR*** ($p < 2 \times 10^{-16}$)	0.337 (1.603×10^{5})	0.282 (1.064×10^{6})	0.275 (5.395×10^{4})	0.313 (7.517×10^{5})	0.286 (4.912×10^{5})	0.308 (1.700×10^{5})
MX*** ($p < 2 \times 10^{-16}$)	0.341 (7.651×10^{3})	0.282 (4.585×10^{5})	0.286 (8.275×10^{5})	0.317 (1.154×10^{5})	0.287 (1.142×10^{6})	0.314 (1.393×10^{5})

Total number of megabases, summed across all individuals, in parentheses. A significant difference (Pearson's chi-square test, p value in parentheses) across populations for a given ancestry background is denoted at the beginning of a column. A significant difference across ancestry backgrounds for a given population (Pearson's chi-square test, p value in parentheses) is denoted at the beginning of a row. Population codes: AA, African American; PR, Puerto Rican; MX, Mexican American. Local ancestry codes: AFR, homozygous African; EUR, homozygous European; NAM, homozygous Native American; EUAF, heterozygous European/African; EUNA, heterozygous European/Native American; AFNA, heterozygous African/Native American. *$p < 0.05$, **$p < 0.01$, ***$p < 0.001$.

more European and Native American ancestry, which tend to have more genome-wide deleterious homozygotes (Figure 3B) as a result of the serial bottlenecks they experienced since migrating out of Africa.

We have already shown (Figure 4) that as total genomic ROH increases the proportion of deleterious homozygotes falling in ROH increases faster than the proportion of benign homozygotes, but here we want to know whether the ancestral background of the IBD haplotypes matters. We propose that haplotypes sourced from ancestral populations with high deleterious heterozygosity have highest rates of accumulation of deleterious homozygotes when paired IBD to generate ROH.

To test this proposition, we first partition ROH based on the ancestral background of the underlying IBD haplotypes. Then we compute for each individual (i) the fraction of all deleterious (d) and benign (b) homozygotes across the genome that fall into each ROH class (j) as:

$$f_{i,j}^{d}(A) = \frac{g_{i,j}^{d,2}(A)}{g_{i,R}^{d,2} + g_{i,N}^{d,2}}$$

and

$$f_{i,j}^{b}(A) = \frac{g_{i,j}^{b,2}(A)}{g_{i,R}^{b,2} + g_{i,N}^{b,2}},$$

where $g_{i,j}^{d,2}(A)$ and $g_{i,j}^{b,2}(A)$ are the number of deleterious and benign homozygotes, respectively, in individual i in ROH class j on ancestral haplotype background $A \in \{AFR, EUR, NAM\}$. Similarly, $f_{i,j}^{d}(A)$ and $f_{i,j}^{b}(A)$ are the genome-wide fraction of deleterious and benign homozygotes, respectively, in individual i in ROH class j that fall on haplotype background A. Finally, we fit a linear model similar as above, $f_{\cdot j}(A) = \beta_0 + \beta_1 G_{\cdot j}(A) + \beta_2 D + \beta_3 D G_{\cdot j}(A) + \varepsilon$, in order to test for differences in the rate of accumulation (β_3) of deleterious homozygotes compared to benign homozygotes as a function of $G_{\cdot j}(A)$, the genomic fraction of ROH on ancestral background A. The results are plotted in Figure 5 for total ROH ($j = N$; Figures 5A–5C) and for long ROH ($j = C$; Figures 5D–5F), and the regression coefficients are also summarized in Table S2.

For total ROH, we find significant differences in the rate of accumulation of deleterious homozygotes on all ancestry backgrounds (Figures 5A–5C). Furthermore, consistent with our expectations, we find that ROH on African ancestral haplotypes have the highest rate difference ($\beta_3 = 1.214$, $p < 2 \times 10^{-16}$; Figure 5C), whereas ROH on European ancestral haplotypes have an intermediate rate difference ($\beta_3 = 0.648$, $p < 2 \times 10^{-16}$; Figure 5B) and ROH on Native American ancestral haplotypes have the lowest rate difference ($\beta_3 = 0.510$, $p < 2 \times 10^{-16}$; Figure 5A). This pattern is repeated when we consider only long ROH comprised of young haplotypes (Figures 5D–5F) and also when we analyze smaller ROH (albeit with weaker effects; Figure S1).

We also perform a variation of this analysis to compare the rate of gain of deleterious homozygotes in high-pLI versus low-pLI genes in ROH across different ancestral backgrounds. We fit the regression $f_{\cdot j}(A) = \beta_0 + \beta_1 G_{\cdot j}(A) + \beta_2 D + \beta_3 D G_{\cdot j}(A) + \varepsilon$, which is similar to above except that H is an indicator variable taking a value of 1 or 0 if the response comes from the high-pLI gene set or the low-pLI gene set, respectively (Table S5).

For all ROH combined, we find a significantly higher rate of gain of deleterious homozygotes in high-pLI genes versus low-pLI genes on Native American haplotypes ($\beta_3 = 0.0746, p = 9.030 \times 10^{-3}$) but not for European ($\beta_3 = 0.0584, p = 0.114$) or African ($\beta_3 = -8.246 \times 10^{-3}, p = 0.852$) haplotypes. Considering only long ROH, there is a significant difference for Native American ($\beta_3 = 0.0973, p = 1.571 \times 10^{-2}$) and European ($\beta_3 = 0.133, p = 2.520 \times 10^{-2}$), but again not for African ($\beta_3 = 0.146, p = 0.122$). Since we have restricted our dataset by gene set, ROH class, and ancestral background, we may lack power to detect small effect sizes in this African case. Alternatively, there may be more complicated dynamics relating deleteriousness to demography and inbreeding.

We next directly compare the rate of increase of deleterious homozygotes across different ancestral haplotype backgrounds. To do this we compute the following regression, $f_{\cdot j}^{d}(\cdot) = \beta_0 + \beta_1 G_{\cdot j}(\cdot) + \beta_2 I(EUR) + \beta_3 I(NAM) + \beta_4 I(EUR) G_{\cdot j}(\cdot) + \beta_5 I(NAM) G_{\cdot j}(\cdot) + \varepsilon$, where $f_{\cdot j}^{d}(\cdot)$ is a

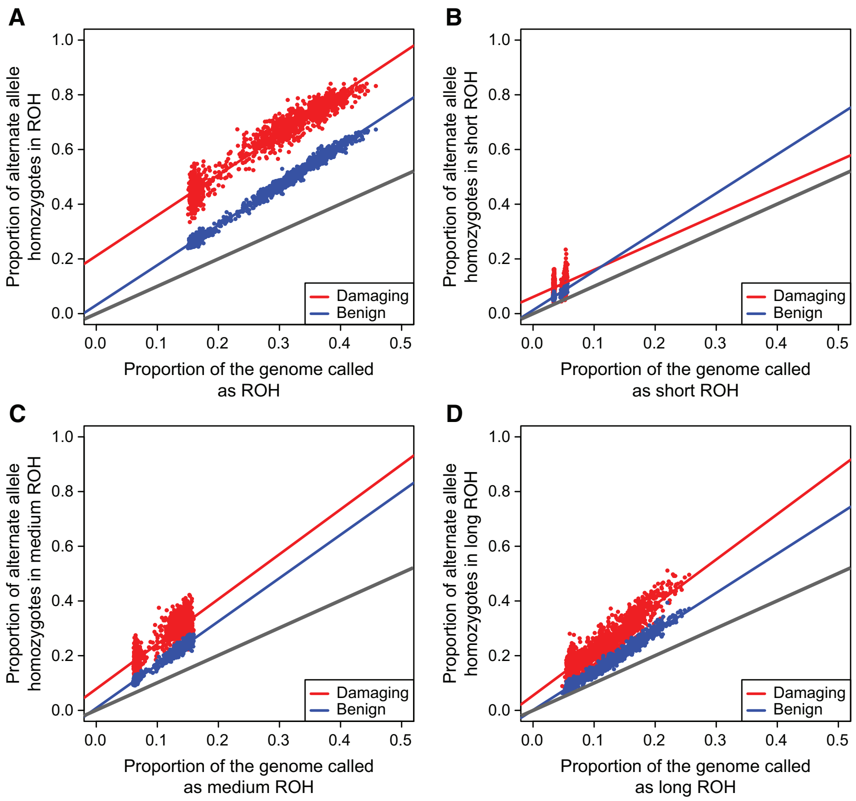

Figure 4. Deleterious and Benign Homozygotes in ROH Classes
The proportion of damaging (red) and benign (blue) homozygotes falling in ROH of different size classes: (A) all ROH, (B) short ROH, (C) medium ROH, and (D) long ROH. Data shown is across all populations. Gray line plots Y = X.

vector representing the proportion of damaging homozygotes in ROH class j on each local ancestry background across all individuals. $G_{\cdot j}(\cdot)$ represents the genome-wide fraction ROH class j falling on each local ancestry background across all individuals, and $I(A)$ is an indicator variable which takes the value 1 if the associated response is on ancestral background $A \in \{AFR, EUR, NAM\}$ and takes the value 0 otherwise. Here we analyze each ROH class: all, long, medium, and short.

We plot the results for "all" and "long" in Figure 6 ("medium" and "short" in Figure S2) and summarize the inferred regression coefficients for all classes in Table S3. We focus on the regression coefficients β_4 and β_5, which represent the difference in rate of gain of deleterious homozygotes in ROH on European or Native American haplotypes compared to African haplotypes, respectively. Graphically, in Figures 6 and S2, a significant β_4 corresponds to a significant difference in the slope of the orange and blue line, and a significant β_5 corresponds to a significant difference in the slope of the orange and red line. Since we expect that the rate of gain of deleterious homozygotes to be lowest in ROH on European and Native American haplotypes compared to ROH on African ones, we expect significant negative values for both β_4 and β_5.

Consistent with our expectations, when analyzing all ROH (Figure 6A) we find a significant negative $\beta_4 = -0.763$ ($p < 2\times 10^{-16}$) and $\beta_5 = -0.852$ ($p < 2\times 10^{-16}$), indicating that the gain rate of damaging homozygotes in ROH on African ancestral haplotypes outpaces that of ROH on the other ancestral haplotypes. This pattern continues when considering only long ROH ($\beta_4 = -0.852$, $p < 2\times10^{-16}$; $\beta_5 = -0.727$, $p < 2\times 10^{-16}$; Figure 6B) and smaller ROH (Table S3 and Figure S2).

We repeat a similar analysis to compare the rate of gain of deleterious homozygotes in high-pLI genes directly across ancestry backgrounds. In this case, although African ancestral backgrounds do not show a significant difference in the accumulation of deleterious homozygotes between high- and low-pLI genes, they show a clearly higher rate of gain in high-pLI genes compared to European and Native American ancestral backgrounds (Table S6).

To check the robustness of these results, we reran these analyses using several other deleterious classification methods including SIFT,[82,93] Provean,[83] and GERP.[84] Since GERP scores sites and not mutations, we restricted the GERP analysis to loci where the ancestral and derived states were inferred to high confidence. As this ancestral polarization results in discarding a large number of loci with ambiguous ancestral allele state, we also reran these analyses for PolyPhen 2,[81] SIFT,[82,93] and Provean[83] restricted only to loci for which we have ancestral/derived state information. Figure S3 plots the inferred $\beta_3$for each of these analyses for each ROH size class and demonstrates qualitatively similar patterns as shown above.

We further re-analyzed a subset of the ROH and deleteriousness calls from Pemberton and Szpiech,[53] which contains data on six admixed populations from the 1000 Genomes Project[94] and used CADD[95] scores as a deleteriousness prediction (Supplemental Material and Methods). After extracting the data relating to the admixed individuals from Pemberton and Szpiech[53] and calling local ancestries, we again find qualitatively similar patterns as above (Figure S4).

Since Pemberton and Szpiech[53] showed that these enrichment patterns appear to be driven by an abundance of homozygotes in ROH comprised of low-frequency alleles, we re-analyzed our data using categories of minor allele frequency (MAF) instead of deleteriousness (see

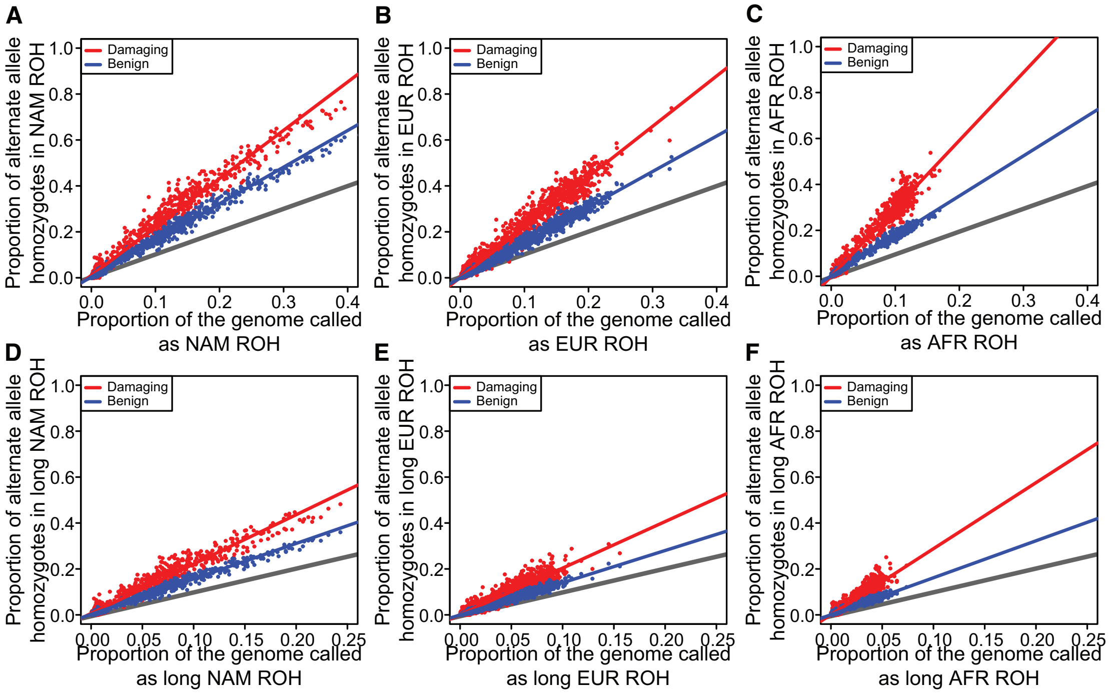

Figure 5. Deleterious and Benign Homozygotes in ROH Classes Separated by Ancestry
The proportion of damaging (red) and benign (blue) homozygotes falling in ROH comprised of different ancestral haplotypes and size classes: (A) all NAM ROH, (B) all EUR ROH, (C) all AFR ROH, (D) long NAM ROH, (E) long EUR ROH, and (F) long AFR ROH. EUR, European; AFR, African, and NAM, Native American. Gray line plots Y = X.

Material and Methods for how we determined MAF category). Using these allele frequencies, we categorize each polymorphic locus in a gene region (exons plus introns) into one of two categories: common (MAF $\geq$ 0.05) and rare (MAF $<$ 0.05). We then fit the same models as above, except that instead of comparing the proportion of deleterious alternate allele homozygotes to benign homozygotes as a function of ROH coverage, we compare the number of minor allele homozygotes in the rare class to the common class.

We summarize the results of these analyses for each ancestral background, each ROH size class, and each low-frequency class in Figure 7. We find that ROH on African haplotype backgrounds gain more low-frequency minor allele homozygotes per unit increase of ROH (and especially long class C ROH) compared to common minor allele homozygotes. Since low-frequency alleles are enriched for deleterious variants relative to high-frequency alleles, this result accords with our previous analyses.

Simulating Deleterious Alleles in ROH

We have proposed that autozygosity of haplotypes with recent ancestry from high-heterozygosity source populations concentrate deleterious homozygotes at a higher rate per unit increase of ROH coverage (Figure 6). We wish to test via simulations whether these differences in ancestral demographic history can account for this pattern. To this end, we simulate recessive deleterious alleles in a complex three population demographic history,[90] corresponding roughly to African, European, and Asian human populations (see Material and Methods). Although our other analyses considered haplotypes from African, European, and Native American ancestral populations, this three-population demographic model has been well studied and is readily available. As this three-population model contains a high-heterozygosity source population with two population splits undergoing multiple bottlenecks, we feel this will provide a set of simulated data with a qualitatively similar demographic history.

For each of 500 simulation replicates, we sample 500 diploid individuals from each population, call ROH, and then compute the proportion of genome-wide deleterious homozygotes falling within each ROH class. We then compute a regression, similar to the previous section where we analyzed the differences between deleterious homozygotes in ROH on different ancestral backgrounds. We compute, $f^d_{\cdot j}(\cdot) = \beta_0 + \beta_1 G_{\cdot j}(\cdot) + \beta_2 I(EUR) + \beta_3 I(ASN) + \beta_4 I(EUR) G_{\cdot j}(\cdot) + \beta_5 I(ASN) G_{\cdot j}(\cdot) + \varepsilon$, where $f^d_{\cdot j}(\cdot)$ is a vector representing the proportion of damaging homozygotes in ROH class j in each population across all individuals. $G_{\cdot j}(\cdot)$ represents the genome-wide fraction ROH class j in each population across all individuals, and $I(A)$ is an indicator variable which takes the value 1 if the associated individual is from population $A \in \{AFR, EUR, ASN\}$ and takes the value 0 otherwise. Here AFR corresponds to the simulated African population, EUR corresponds to the simulated European population, and ASN corresponds to the simulated Asian population. We analyze each ROH class: all, long, medium, and short, and within each class we combine our

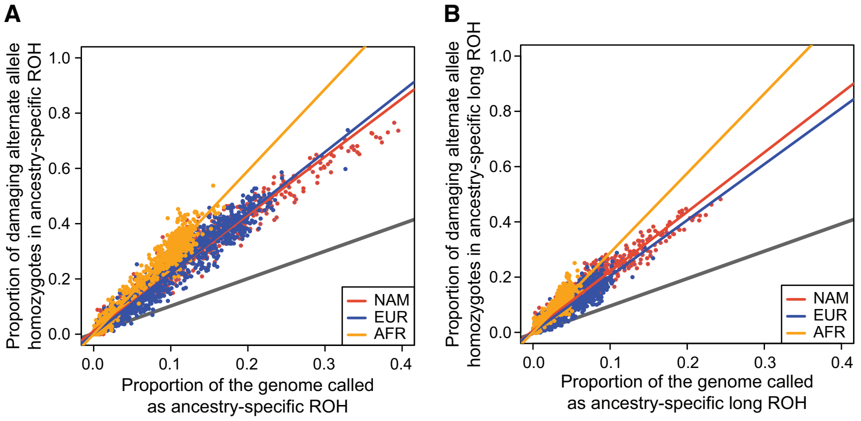

Figure 6. Deleterious Homozygotes in ROH Classes Compared across Ancestry
A direct comparison of the proportion of damaging homozygotes falling in ROH comprised of different ancestral haplotypes for (A) all ROH and (B) long ROH. EUR, European, colored blue; AFR, African, colored orange; and NAM, Native American, colored red. Gray line plots Y = X.

regression coefficients across replicates with inverse-variance weighted meta-analysis.

In this formulation, the regression terms β_4 and β_5 represent the difference in rate of gain of deleterious homozygotes in ROH on European or Asian haplotypes compared to African haplotypes, respectively. For example, a $\beta_4 = 1$ would represent a scenario where an increase of 1% ROH genome-wide in the simulated European population concentrated 1% more genome-wide deleterious homozygotes in those regions compared to the simulated African population. Similarly, a $\beta_5 = -1$ would represent a scenario where an increase of 1% ROH genome-wide in the simulated Asian population concentrated 1% less genome-wide deleterious homozygotes in those regions compared to the simulated African population. Since we hypothesize that the simulated African population will have the highest rate of gain of deleterious homozygotes as a function of genomic ROH coverage, we expect both of these terms to be negative. Indeed, this is what we find across all ROH classes (Table S7). Considering all ROH together, we find $\beta_4 = -0.409$ ($p < 2 \times 10^{-16}$) and $\beta_5 = -0.488$ ($p < 2 \times 10^{-16}$), and when analyzing only long ROH we find $\beta_4 = -0.386$ ($p < 2 \times 10^{-16}$) and $\beta_5 = -0.446$ ($p < 2 \times 10^{-16}$).

Discussion

The distribution of runs of homozygosity in individual genomes has provided insights into evolutionary, population, and medical genetics.[1] By examining their genomic location and prevalence in a population, we can learn about the history and adaptation of natural populations,[2–30,96,97] and we can make discoveries about the genetic basis of complex phenotypes.[32–48] Given the importance of demographic history and socio-cultural practices in the generation of ROH in individual genomes, and their relationship to complex phenotypes including many genetic diseases, it naturally follows to study the distribution of deleterious alleles and their relationship to ROH.

Previous work has described the effect of demographic history on the distribution of deleterious alleles,[31,70–73,98] including a few specifically investigating their relationship with runs of homozygosity.[17,29,31,52,53,99,100] However, little work has been done on the relationship between deleterious alleles and ROH in admixed populations (although see Mooney et al.[100]). Since there is evidence of very recent bottlenecks (which generate ROH) within admixed populations living in the Americas,[63,100] the relationship between ROH and the accumulation of deleterious homozygotes may provide valuable insights into the genetic basis of complex phenotypes in these individuals.

Here we analyzed 1,441 individuals across three admixed populations: African American, Puerto Rican, and Mexican American. We found that, consistent with other studies, the proportion of deleterious homozygotes found in ROH increases faster than the proportion of benign homozygotes as a function of total genomic ROH (Figure 4 and Table S1). We also found that the genome-wide proportion of deleterious homozygotes in ROH on African ancestral haplotypes increased faster per unit ROH than on ether European or Native American ancestral haplotypes (Figures 5, 6, and Tables S2 and S3). These patterns are also consistent with population-specific worldwide patterns of deleterious homozygotes in ROH,[53] where three of the five African populations analyzed had among the highest rates of enrichment in long ROH.

To explain this observation, we propose that ancestral haplotypes from populations with high deleterious *heterozygosity* would exhibit even greater increases of deleterious homozygotes per unit ROH. We reason that, under random mating, the larger number of low-frequency deleterious alleles in the population would largely segregate as heterozygotes, whereas, when a harsh bottleneck or consanguinity occurs, these mutations get paired IBD as homozygotes, concentrating more deleterious homozygotes within ROH. Indeed, via simulation of a realistic human demographic history, we found that the rate of gain of deleterious homozygotes was significantly higher in high heterozygosity source populations compared to others (Table S7).

The idea that population bottlenecks and inbreeding can concentrate more deleterious homozygotes on haplotype backgrounds from a high heterozygosity founder population has also been proposed as a reason for the deterioration of the wolf population on Isle

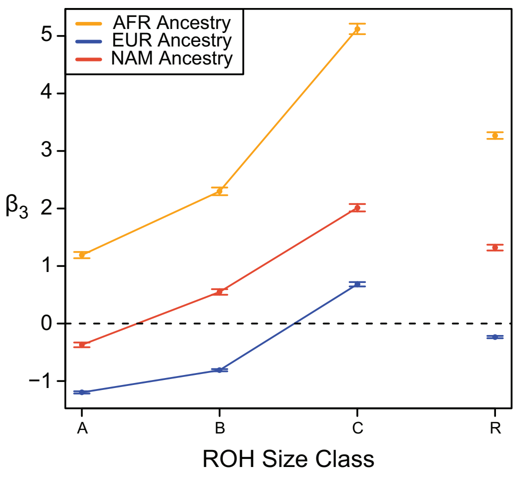

Figure 7. Enrichment of Low-Frequency Variants across ROH Sizes
The difference in rate of gain of low-frequency minor allele homozygotes (MAF < 0.05) compared to common minor allele homozygotes (MAF ≥ 0.05; β_3 from regression analysis). ROH size classes: A, short; B, medium; C, long; R, all sizes. EUR, European, colored blue; AFR, African, colored orange; and NAM, Native American, colored red. Error bars represent standard error of the regression coefficient.

Royale, MI, USA.[31] This population, numbering around 50 at its height, was founded by two to three animals from a large and genetically diverse source population on mainland Minnesota. The extreme bottleneck and inbreeding have manifested numerous conspicuous phenotypes among these wolves, and several extremely long ROH have been identified in its members. This can be contrasted with the historically small wolf populations in Ethiopia, which have successfully avoided the pitfalls of inbreeding depression. Robinson et al.[31] further demonstrate through simulations that although historically small populations tend to have a higher burden of deleterious alleles, there are fewer strongly deleterious alleles segregating compared to large populations. Thus, in the event of a population size crash or inbreeding, smaller populations have reduced risk of severe fitness consequences compared to large populations.

This suggests that ROH on haplotypes from high-heterozygosity populations (e.g., African populations) may generate more homozygotes of strong deleterious alleles compared to other haplotype backgrounds. In the context of human health, this may mean that ROH on those haplotype backgrounds are relevant for understanding the genetic basis of various diseases.

Whereas ROH on any haplotype background are associated with an increased rate of deleterious homozygotes, we show that ROH on African haplotypes tend to have a larger share of the genome-wide deleterious homozygotes. Indeed, this accords with recent work that has independently associated increased ROH[47] and increased African ancestry[74] with reduced lung function. This suggests that these ROH on African haplotypes may play a particularly important role in the genetic architecture of complex phenotypes in admixed individuals, especially for populations with African ancestry that have undergone very harsh bottlenecks in the recent past.

Supplemental Data

Supplemental Data can be found online at https://doi.org/10.1016/j.ajhg.2019.08.011.

Acknowledgments

Support to Z.A.S. and R.D.H. was provided by the National Human Genome Research Institute of the National Institutes of Health (R01HG007644). M.J.W. was supported in part by grant K01HL140218. A.C.Y.M. was supported in part by grants R01MD010443, U01HG009080, R01HL128439, R01HL135156, R01HL141992, R01HL141845, and R01HL117004. E.G.B. was supported in part by grants R01HL128439, R01HL135156, R01ES015794, R21ES24844, P60MD006902, R01MD010443, RL5GM118984, 24RT-0025, Sandler Family Foundation, American Asthma Foundation, RWJF Amos Medical Faculty Development Program, and Harry Wm. and Diana V. Hind Distinguished Professor in Pharmaceutical Sciences II.

Whole-genome sequencing (WGS) for the Trans-Omics in Precision Medicine (TOPMed) program was supported by the National Heart, Lung, and Blood Institute (NHLBI). WGS for "NHLBI TOPMed: Genes-environments & Admixture in Latino Americans (GALA II) Study" (phs000920) and "NHLBI TOPMed: Study of African Americans, Asthma, Genes and Environments (SAGE)" (phs000921) was performed at the New York Genome Center (3R01HL117004-01S3). See Supplemental Acknowledgments for a list of all study collaborators and recruiters.

We gratefully acknowledge the studies and participants who provided biological samples and data for TOPMed, the contributions of the investigators of the NHLBI TOPMed Consortium (https://www.nhlbiwgs.org/topmed-banner-authorship), and the comments of two anonymous reviewers.

Declaration of Interests

The authors declare no competing interests.

Received: March 6, 2019
Accepted: August 27, 2019
Published: September 19, 2019

Web Resources

Axiom Genotype Dataset, https://www.thermofisher.com/us/en/home/life-science/microarray-analysis/microarray-data-analysis/microarray-analysis-sample-data/axiom-genotype-data-set.html
dbGaP, https://www.ncbi.nlm.nih.gov/gap
gnomAD database, https://gnomad.broadinstitute.org/downloads/
ROH simulation code, https://github.com/szpiech/ROHsims
WGSA pre-computed annotations, http://web.corral.tacc.utexas.edu/WGSAdownload/resources/precomputed/

References

1. Ceballos, F.C., Joshi, P.K., Clark, D.W., Ramsay, M., and Wilson, J.F. (2018). Runs of homozygosity: windows into population history and trait architecture. Nat. Rev. Genet. *19*, 220–234.
2. McQuillan, R., Leutenegger, A.L., Abdel-Rahman, R., Franklin, C.S., Pericic, M., Barac-Lauc, L., Smolej-Narancic, N., Janicijevic, B., Polasek, O., Tenesa, A., et al. (2008). Runs of homozygosity in European populations. Am. J. Hum. Genet. *83*, 359–372.
3. Kirin, M., McQuillan, R., Franklin, C.S., Campbell, H., McKeigue, P.M., and Wilson, J.F. (2010). Genomic runs of homozygosity record population history and consanguinity. PLoS ONE *5*, e13996.
4. Pemberton, T.J., Absher, D., Feldman, M.W., Myers, R.M., Rosenberg, N.A., and Li, J.Z. (2012). Genomic patterns of homozygosity in worldwide human populations. Am. J. Hum. Genet. *91*, 275–292.
5. Blant, A., Kwong, M., Szpiech, Z.A., and Pemberton, T.J. (2017). Weighted likelihood inference of genomic autozygosity patterns in dense genotype data. BMC Genomics *18*, 928.
6. Kardos, M., Luikart, G., and Allendorf, F.W. (2015). Measuring individual inbreeding in the age of genomics: marker-based measures are better than pedigrees. Heredity *115*, 63–72.
7. Mastrangelo, S., Tolone, M., Di Gerlando, R., Fontanesi, L., Sardina, M.T., and Portolano, B. (2016). Genomic inbreeding estimation in small populations: evaluation of runs of homozygosity in three local dairy cattle breeds. Animal *10*, 746–754.
8. Kang, J.T.L., Goldberg, A., Edge, M.D., Behar, D.M., and Rosenberg, N.A. (2016). Consanguinity Rates Predict Long Runs of Homozygosity in Jewish Populations. Hum. Hered. *82*, 87–102.
9. Meyer, M., Kircher, M., Gansauge, M.T., Li, H., Racimo, F., Mallick, S., Schraiber, J.G., Jay, F., Prüfer, K., de Filippo, C., et al. (2012). A high-coverage genome sequence from an archaic Denisovan individual. Science *338*, 222–226.
10. Castellano, S., Parra, G., Sánchez-Quinto, F.A., Racimo, F., Kuhlwilm, M., Kircher, M., Sawyer, S., Fu, Q., Heinze, A., Nickel, B., et al. (2014). Patterns of coding variation in the complete exomes of three Neandertals. Proc. Natl. Acad. Sci. USA *111*, 6666–6671.
11. Prüfer, K., Racimo, F., Patterson, N., Jay, F., Sankararaman, S., Sawyer, S., Heinze, A., Renaud, G., Sudmant, P.H., de Filippo, C., et al. (2014). The complete genome sequence of a Neanderthal from the Altai Mountains. Nature *505*, 43–49.
12. Gamba, C., Jones, E.R., Teasdale, M.D., McLaughlin, R.L., Gonzalez-Fortes, G., Mattiangeli, V., Domboróczki, L., Kővári, I., Pap, I., Anders, A., et al. (2014). Genome flux and stasis in a five millennium transect of European prehistory. Nat. Commun. *5*, 5257.
13. Prado-Martinez, J., Sudmant, P.H., Kidd, J.M., Li, H., Kelley, J.L., Lorente-Galdos, B., Veeramah, K.R., Woerner, A.E., O'Connor, T.D., Santpere, G., et al. (2013). Great ape genetic diversity and population history. Nature *499*, 471–475.
14. Xue, Y., Prado-Martinez, J., Sudmant, P.H., Narasimhan, V., Ayub, Q., Szpak, M., Frandsen, P., Chen, Y., Yngvadottir, B., Cooper, D.N., et al. (2015). Mountain gorilla genomes reveal the impact of long-term population decline and inbreeding. Science *348*, 242–245.
15. Palkopoulou, E., Mallick, S., Skoglund, P., Enk, J., Rohland, N., Li, H., Omrak, A., Vartanyan, S., Poinar, H., Götherström, A., et al. (2015). Complete genomes reveal signatures of demographic and genetic declines in the woolly mammoth. Curr. Biol. *25*, 1395–1400.
16. Curik, I., Ferencakovic, M., and Solkner, J. (2014). Inbreeding and runs of homozygosity: A possible solution to an old problem. Livest. Sci. *166*, 26–34.
17. Zhang, Q., Guldbrandtsen, B., Bosse, M., Lund, M.S., and Sahana, G. (2015). Runs of homozygosity and distribution of functional variants in the cattle genome. BMC Genomics *16*, 542.
18. Manunza, A., Noce, A., Serradilla, J.M., Goyache, F., Martínez, A., Capote, J., Delgado, J.V., Jordana, J., Muñoz, E., Molina, A., et al. (2016). A genome-wide perspective about the diversity and demographic history of seven Spanish goat breeds. Genet. Sel. Evol. *48*, 52.
19. Gurgul, A., Szmatoła, T., Topolski, P., Jasielczuk, I., Żukowski, K., and Bugno-Poniewierska, M. (2016). The use of runs of homozygosity for estimation of recent inbreeding in Holstein cattle. J. Appl. Genet. *57*, 527–530.
20. Peripolli, E., Stafuzza, N.B., Munari, D.P., Lima, A.L.F., Irgang, R., Machado, M.A., Panetto, J.C.D.C., Ventura, R.V., Baldi, F., and da Silva, M.V.G.B. (2018). Assessment of runs of homozygosity islands and estimates of genomic inbreeding in Gyr (Bos indicus) dairy cattle. BMC Genomics *19*, 34.
21. Forutan, M., Ansari Mahyari, S., Baes, C., Melzer, N., Schenkel, F.S., and Sargolzaei, M. (2018). Inbreeding and runs of homozygosity before and after genomic selection in North American Holstein cattle. BMC Genomics *19*, 98.
22. Kardos, M., Qvarnström, A., and Ellegren, H. (2017). Inferring Individual Inbreeding and Demographic History from Segments of Identity by Descent in *Ficedula* Flycatcher Genome Sequences. Genetics *205*, 1319–1334.
23. Bortoluzzi, C., Crooijmans, R.P.M.A., Bosse, M., Hiemstra, S.J., Groenen, M.A.M., and Megens, H.J. (2018). The effects of recent changes in breeding preferences on maintaining traditional Dutch chicken genomic diversity. Heredity *121*, 564–578.
24. Bertolini, F., Gandolfi, B., Kim, E.S., Haase, B., Lyons, L.A., and Rothschild, M.F. (2016). Evidence of selection signatures that shape the Persian cat breed. Mamm. Genome *27*, 144–155.
25. vonHoldt, B.M., Pollinger, J.P., Earl, D.A., Knowles, J.C., Boyko, A.R., Parker, H., Geffen, E., Pilot, M., Jedrzejewski, W., Jedrzejewska, B., et al. (2011). A genome-wide perspective on the evolutionary history of enigmatic wolf-like canids. Genome Res. *21*, 1294–1305.
26. Friedenberg, S.G., Meurs, K.M., and Mackay, T.F.C. (2016). Evaluation of artificial selection in Standard Poodles using whole-genome sequencing. Mamm. Genome *27*, 599–609.
27. Metzger, J., Pfahler, S., and Distl, O. (2016). Variant detection and runs of homozygosity in next generation sequencing data elucidate the genetic background of Lundehund syndrome. BMC Genomics *17*, 535.
28. Dreger, D.L., Davis, B.W., Cocco, R., Sechi, S., Di Cerbo, A., Parker, H.G., Polli, M., Marelli, S.P., Crepaldi, P., and Ostrander, E.A. (2016). Commonalities in Development of Pure Breeds and Population Isolates Revealed in the Genome of the Sardinian Fonni's Dog. Genetics *204*, 737–755.

29. Sams, A.J., and Boyko, A.R. (2019). Fine-Scale Resolution of Runs of Homozygosity Reveal Patterns of Inbreeding and Substantial Overlap with Recessive Disease Genotypes in Domestic Dogs. G3 (Bethesda) *9*, 117–123.
30. Kardos, M., Åkesson, M., Fountain, T., Flagstad, Ø., Liberg, O., Olason, P., Sand, H., Wabakken, P., Wikenros, C., and Ellegren, H. (2018). Genomic consequences of intensive inbreeding in an isolated wolf population. Nat. Ecol. Evol. *2*, 124–131.
31. Robinson, J.A., Räikkönen, J., Vucetich, L.M., Vucetich, J.A., Peterson, R.O., Lohmueller, K.E., and Wayne, R.K. (2019). Genomic signatures of extensive inbreeding in Isle Royale wolves, a population on the threshold of extinction. Sci. Adv. *5*, u0757.
32. Johnson, E.C., Evans, L.M., and Keller, M.C. (2018). Relationships between estimated autozygosity and complex traits in the UK Biobank. PLoS Genet. *14*, e1007556.
33. Sheridan, E., Wright, J., Small, N., Corry, P.C., Oddie, S., Whibley, C., Petherick, E.S., Malik, T., Pawson, N., McKinney, P.A., and Parslow, R.C. (2013). Risk factors for congenital anomaly in a multiethnic birth cohort: an analysis of the Born in Bradford study. Lancet *382*, 1350–1359.
34. Scott, E.M., Halees, A., Itan, Y., Spencer, E.G., He, Y., Azab, M.A., Gabriel, S.B., Belkadi, A., Boisson, B., Abel, L., et al.; Greater Middle East Variome Consortium (2016). Characterization of Greater Middle Eastern genetic variation for enhanced disease gene discovery. Nat. Genet. *48*, 1071–1076.
35. Ismail, J., Jafar, T.H., Jafary, F.H., White, F., Faruqui, A.M., and Chaturvedi, N. (2004). Risk factors for non-fatal myocardial infarction in young South Asian adults. Heart *90*, 259–263.
36. Christofidou, P., Nelson, C.P., Nikpay, M., Qu, L., Li, M., Loley, C., Debiec, R., Braund, P.S., Denniff, M., Charchar, F.J., et al. (2015). Runs of Homozygosity: Association with Coronary Artery Disease and Gene Expression in Monocytes and Macrophages. Am. J. Hum. Genet. *97*, 228–237.
37. Simpson, J.L., Martin, A.O., Elias, S., Sarto, G.E., and Dunn, J.K. (1981). Cancers of the breast and female genital system: search for recessive genetic factors through analysis of human isolate. Am. J. Obstet. Gynecol. *141*, 629–636.
38. Rudan, I. (1999). Inbreeding and cancer incidence in human isolates. Hum. Biol. *71*, 173–187.
39. Bacolod, M.D., Schemmann, G.S., Wang, S., Shattock, R., Giardina, S.F., Zeng, Z., Shia, J., Stengel, R.F., Gerry, N., Hoh, J., et al. (2008). The signatures of autozygosity among patients with colorectal cancer. Cancer Res. *68*, 2610–2621.
40. Rudan, I., Smolej-Narancic, N., Campbell, H., Carothers, A., Wright, A., Janicijevic, B., and Rudan, P. (2003). Inbreeding and the genetic complexity of human hypertension. Genetics *163*, 1011–1021.
41. Campbell, H., Carothers, A.D., Rudan, I., Hayward, C., Biloglav, Z., Barac, L., Pericic, M., Janicijevic, B., Smolej-Narancic, N., Polasek, O., et al. (2007). Effects of genome-wide heterozygosity on a range of biomedically relevant human quantitative traits. Hum. Mol. Genet. *16*, 233–241.
42. Keller, M.C., Simonson, M.A., Ripke, S., Neale, B.M., Gejman, P.V., Howrigan, D.P., Lee, S.H., Lencz, T., Levinson, D.F., Sullivan, P.F.; and Schizophrenia Psychiatric Genome-Wide Association Study Consortium (2012). Runs of homozygosity implicate autozygosity as a schizophrenia risk factor. PLoS Genet. *8*, e1002656.
43. Gandin, I., Faletra, F., Faletra, F., Carella, M., Pecile, V., Ferrero, G.B., Biamino, E., Palumbo, P., Palumbo, O., Bosco, P., et al. (2015). Excess of runs of homozygosity is associated with severe cognitive impairment in intellectual disability. Genet. Med. *17*, 396–399.
44. Mukherjee, S., Guha, S., Ikeda, M., Iwata, N., Malhotra, A.K., Pe'er, I., Darvasi, A., and Lencz, T. (2014). Excess of homozygosity in the major histocompatibility complex in schizophrenia. Hum. Mol. Genet. *23*, 6088–6095.
45. Ghani, M., Reitz, C., Cheng, R., Vardarajan, B.N., Jun, G., Sato, C., Naj, A., Rajbhandary, R., Wang, L.S., Valladares, O., et al.; Alzheimer's Disease Genetics Consortium (2015). Association of Long Runs of Homozygosity With Alzheimer Disease Among African American Individuals. JAMA Neurol. *72*, 1313–1323.
46. McQuillan, R., Eklund, N., Pirastu, N., Kuningas, M., McEvoy, B.P., Esko, T., Corre, T., Davies, G., Kaakinen, M., Lyytikäinen, L.P., et al.; ROHgen Consortium (2012). Evidence of inbreeding depression on human height. PLoS Genet. *8*, e1002655.
47. Joshi, P.K., Esko, T., Mattsson, H., Eklund, N., Gandin, I., Nutile, T., Jackson, A.U., Schurmann, C., Smith, A.V., Zhang, W., et al. (2015). Directional dominance on stature and cognition in diverse human populations. Nature *523*, 459–462.
48. Lyons, E.J., Frodsham, A.J., Zhang, L., Hill, A.V., and Amos, W. (2009). Consanguinity and susceptibility to infectious diseases in humans. Biol. Lett. *5*, 574–576.
49. Pritchard, J.K., and Cox, N.J. (2002). The allelic architecture of human disease genes: common disease-common variant...or not? Hum. Mol. Genet. *11*, 2417–2423.
50. Carlson, C.S., Eberle, M.A., Kruglyak, L., and Nickerson, D.A. (2004). Mapping complex disease loci in whole-genome association studies. Nature *429*, 446–452.
51. Boyle, E.A., Li, Y.I., and Pritchard, J.K. (2017). An Expanded View of Complex Traits: From Polygenic to Omnigenic. Cell *169*, 1177–1186.
52. Szpiech, Z.A., Xu, J., Pemberton, T.J., Peng, W., Zöllner, S., Rosenberg, N.A., and Li, J.Z. (2013). Long runs of homozygosity are enriched for deleterious variation. Am. J. Hum. Genet. *93*, 90–102.
53. Pemberton, T.J., and Szpiech, Z.A. (2018). Relationship between Deleterious Variation, Genomic Autozygosity, and Disease Risk: Insights from The 1000 Genomes Project. Am. J. Hum. Genet. *102*, 658–675.
54. Colby, S.L., and Ortman, J.M. (2014). Projections of the size and composition of the US population: 2014 to 2060, Current Population Reports (Washington, DC: US Census Bureau), pp. 25–1143.
55. Martin, A.R., Gignoux, C.R., Walters, R.K., Wojcik, G.L., Neale, B.M., Gravel, S., Daly, M.J., Bustamante, C.D., and Kenny, E.E. (2017). Human Demographic History Impacts Genetic Risk Prediction across Diverse Populations. Am. J. Hum. Genet. *100*, 635–649.
56. Verdu, P., Austerlitz, F., Estoup, A., Vitalis, R., Georges, M., Théry, S., Froment, A., Le Bomin, S., Gessain, A., Hombert, J.M., et al. (2009). Origins and genetic diversity of pygmy hunter-gatherers from Western Central Africa. Curr. Biol. *19*, 312–318.
57. Via, M., Gignoux, C.R., Roth, L.A., Fejerman, L., Galanter, J., Choudhry, S., Toro-Labrador, G., Viera-Vera, J., Oleksyk, T.K., Beckman, K., et al. (2011). History shaped the geographic

distribution of genomic admixture on the island of Puerto Rico. PLoS ONE *6*, e16513.

58. Moreno-Estrada, A., Gravel, S., Zakharia, F., McCauley, J.L., Byrnes, J.K., Gignoux, C.R., Ortiz-Tello, P.A., Martínez, R.J., Hedges, D.J., Morris, R.W., et al. (2013). Reconstructing the population genetic history of the Caribbean. PLoS Genet. *9*, e1003925.
59. Gravel, S., Zakharia, F., Moreno-Estrada, A., Byrnes, J.K., Muzzio, M., Rodriguez-Flores, J.L., Kenny, E.E., Gignoux, C.R., Maples, B.K., Guiblet, W., et al.; 1000 Genomes Project (2013). Reconstructing Native American migrations from whole-genome and whole-exome data. PLoS Genet. *9*, e1004023.
60. Verdu, P., Pemberton, T.J., Laurent, R., Kemp, B.M., Gonzalez-Oliver, A., Gorodezky, C., Hughes, C.E., Shattuck, M.R., Petzelt, B., Mitchell, J., et al. (2014). Patterns of admixture and population structure in native populations of Northwest North America. PLoS Genet. *10*, e1004530.
61. Homburger, J.R., Moreno-Estrada, A., Gignoux, C.R., Nelson, D., Sanchez, E., Ortiz-Tello, P., Pons-Estel, B.A., Acevedo-Vasquez, E., Miranda, P., Langefeld, C.D., et al. (2015). Genomic Insights into the Ancestry and Demographic History of South America. PLoS Genet. *11*, e1005602.
62. Baharian, S., Barakatt, M., Gignoux, C.R., Shringarpure, S., Errington, J., Blot, W.J., Bustamante, C.D., Kenny, E.E., Williams, S.M., Aldrich, M.C., and Gravel, S. (2016). The Great Migration and African-American Genomic Diversity. PLoS Genet. *12*, e1006059.
63. Browning, S.R., Browning, B.L., Daviglus, M.L., Durazo-Arvizu, R.A., Schneiderman, N., Kaplan, R.C., and Laurie, C.C. (2018). Ancestry-specific recent effective population size in the Americas. PLoS Genet. *14*, e1007385.
64. Zhu, X., Tang, H., and Risch, N. (2008). Admixture mapping and the role of population structure for localizing disease genes. Adv. Genet. *60*, 547–569.
65. Cheng, C.Y., Kao, W.H., Patterson, N., Tandon, A., Haiman, C.A., Harris, T.B., Xing, C., John, E.M., Ambrosone, C.B., Brancati, F.L., et al. (2009). Admixture mapping of 15,280 African Americans identifies obesity susceptibility loci on chromosomes 5 and X. PLoS Genet. *5*, e1000490.
66. Torgerson, D.G., Gignoux, C.R., Galanter, J.M., Drake, K.A., Roth, L.A., Eng, C., Huntsman, S., Torres, R., Avila, P.C., Chapela, R., et al. (2012). Case-control admixture mapping in Latino populations enriches for known asthma-associated genes. J. Allergy Clin. Immunol. *130*, 76–82.e12.
67. Galanter, J.M., Gignoux, C.R., Torgerson, D.G., Roth, L.A., Eng, C., Oh, S.S., Nguyen, E.A., Drake, K.A., Huntsman, S., Hu, D., et al. (2014). Genome-wide association study and admixture mapping identify different asthma-associated loci in Latinos: the Genes-environments & Admixture in Latino Americans study. J. Allergy Clin. Immunol. *134*, 295–305.
68. Spear, M.L., Hu, D., Pino-Yanes, M., Huntsman, S., Eng, C., Levin, A.M., Ortega, V.E., White, M.J., McGarry, M.E., Thakur, N., et al. (2019). A genome-wide association and admixture mapping study of bronchodilator drug response in African Americans with asthma. Pharmacogenomics J. *19*, 249–259.
69. Mak, A.C.Y., White, M.J., Eckalbar, W.L., Szpiech, Z.A., Oh, S.S., Pino-Yanes, M., Hu, D., Goddard, P., Huntsman, S., Galanter, J., et al.; NHLBI Trans-Omics for Precision Medicine (TOPMed) Consortium (2018). Whole-Genome Sequencing of Pharmacogenetic Drug Response in Racially Diverse Children with Asthma. Am. J. Respir. Crit. Care Med. *197*, 1552–1564.
70. Lohmueller, K.E., Indap, A.R., Schmidt, S., Boyko, A.R., Hernandez, R.D., Hubisz, M.J., Sninsky, J.J., White, T.J., Sunyaev, S.R., Nielsen, R., et al. (2008). Proportionally more deleterious genetic variation in European than in African populations. Nature *451*, 994–997.
71. Tennessen, J.A., Bigham, A.W., O'Connor, T.D., Fu, W., Kenny, E.E., Gravel, S., McGee, S., Do, R., Liu, X., Jun, G., et al.; Broad GO; Seattle GO; and NHLBI Exome Sequencing Project (2012). Evolution and functional impact of rare coding variation from deep sequencing of human exomes. Science *337*, 64–69.
72. Fu, W., O'Connor, T.D., Jun, G., Kang, H.M., Abecasis, G., Leal, S.M., Gabriel, S., Rieder, M.J., Altshuler, D., Shendure, J., et al.; NHLBI Exome Sequencing Project (2013). Analysis of 6,515 exomes reveals the recent origin of most human protein-coding variants. Nature *493*, 216–220.
73. Henn, B.M., Botigué, L.R., Bustamante, C.D., Clark, A.G., and Gravel, S. (2015). Estimating the mutation load in human genomes. Nat. Rev. Genet. *16*, 333–343.
74. Pino-Yanes, M., Thakur, N., Gignoux, C.R., Galanter, J.M., Roth, L.A., Eng, C., Nishimura, K.K., Oh, S.S., Vora, H., Huntsman, S., et al. (2015). Genetic ancestry influences asthma susceptibility and lung function among Latinos. J. Allergy Clin. Immunol. *135*, 228–235.
75. Baran, Y., Pasaniuc, B., Sankararaman, S., Torgerson, D.G., Gignoux, C., Eng, C., Rodriguez-Cintron, W., Chapela, R., Ford, J.G., Avila, P.C., et al. (2012). Fast and accurate inference of local ancestry in Latino populations. Bioinformatics *28*, 1359–1367.
76. Kumar, R., Nguyen, E.A., Roth, L.A., Oh, S.S., Gignoux, C.R., Huntsman, S., Eng, C., Moreno-Estrada, A., Sandoval, K., Peñaloza-Espinosa, R.I., et al. (2013). Factors associated with degree of atopy in Latino children in a nationwide pediatric sample: the Genes-environments and Admixture in Latino Asthmatics (GALA II) study. J. Allergy Clin. Immunol. *132*, 896–905.e1.
77. Delaneau, O., Zagury, J.F., and Marchini, J. (2013). Improved whole-chromosome phasing for disease and population genetic studies. Nat. Methods *10*, 5–6.
78. Maples, B.K., Gravel, S., Kenny, E.E., and Bustamante, C.D. (2013). RFMix: a discriminative modeling approach for rapid and robust local-ancestry inference. Am. J. Hum. Genet. *93*, 278–288.
79. Szpiech, Z.A., Blant, A., and Pemberton, T.J. (2017). GARLIC: Genomic Autozygosity Regions Likelihood-based Inference and Classification. Bioinformatics *33*, 2059–2062.
80. Liu, X., White, S., Peng, B., Johnson, A.D., Brody, J.A., Li, A.H., Huang, Z., Carroll, A., Wei, P., Gibbs, R., et al. (2016). WGSA: an annotation pipeline for human genome sequencing studies. J. Med. Genet. *53*, 111–112.
81. Adzhubei, I.A., Schmidt, S., Peshkin, L., Ramensky, V.E., Gerasimova, A., Bork, P., Kondrashov, A.S., and Sunyaev, S.R. (2010). A method and server for predicting damaging missense mutations. Nat. Methods *7*, 248–249.
82. Kumar, P., Henikoff, S., and Ng, P.C. (2009). Predicting the effects of coding non-synonymous variants on protein function using the SIFT algorithm. Nat. Protoc. *4*, 1073–1081.

83. Choi, Y., and Chan, A.P. (2015). PROVEAN web server: a tool to predict the functional effect of amino acid substitutions and indels. Bioinformatics *31*, 2745–2747.
84. Cooper, G.M., Stone, E.A., Asimenos, G., Green, E.D., Batzoglou, S., Sidow, A.; and NISC Comparative Sequencing Program (2005). Distribution and intensity of constraint in mammalian genomic sequence. Genome Res. *15*, 901–913.
85. Karczewski, K.J., Francioli, L.C., Tiao, G., Cummings, B.B., Alföldi, J., Wang, Q., Collins, R.L., Laricchia, K.M., Ganna, A., Birnbaum, D.P., et al. (2019). Variation across 141,456 human exomes and genomes reveals the spectrum of loss-of-function intolerance across human protein-coding genes. bioRxiv. https://doi.org/10.1101/531210.
86. Taliun, D., Harris, D.N., Kessler, M.D., Carlson, J., Szpiech, Z.A., Torres, R., Taliun, S.A.G., Corvelo, A., Gogarten, S.M., Kang, H.M., et al. (2019). Sequencing of 53,831 diverse genomes from the NHLBI TOPMed Program. bioRxiv. https://doi.org/10.1101/563866.
87. Haller, B.C., and Messer, P.W. (2019). SLiM 3: Forward Genetic Simulations Beyond the Wright-Fisher Model. Mol. Biol. Evol. *36*, 632–637.
88. Haller, B.C., Galloway, J., Kelleher, J., Messer, P.W., and Ralph, P.L. (2019). Tree-sequence recording in SLiM opens new horizons for forward-time simulation of whole genomes. Mol. Ecol. Resour. *19*, 552–566.
89. Kelleher, J., Etheridge, A.M., and McVean, G. (2016). Efficient Coalescent Simulation and Genealogical Analysis for Large Sample Sizes. PLoS Comput. Biol. *12*, e1004842.
90. Gravel, S., Henn, B.M., Gutenkunst, R.N., Indap, A.R., Marth, G.T., Clark, A.G., Yu, F., Gibbs, R.A., Bustamante, C.D.; and 1000 Genomes Project (2011). Demographic history and rare allele sharing among human populations. Proc. Natl. Acad. Sci. USA *108*, 11983–11988.
91. Frazer, K.A., Ballinger, D.G., Cox, D.R., Hinds, D.A., Stuve, L.L., Gibbs, R.A., Belmont, J.W., Boudreau, A., Hardenbol, P., Leal, S.M., et al.; International HapMap Consortium (2007). A second generation human haplotype map of over 3.1 million SNPs. Nature *449*, 851–861.
92. Mi, H., Muruganujan, A., Huang, X., Ebert, D., Mills, C., Guo, X., and Thomas, P.D. (2019). Protocol Update for large-scale genome and gene function analysis with the PANTHER classification system (v.14.0). Nat. Protoc. *14*, 703–721.
93. Ng, P.C., and Henikoff, S. (2003). SIFT: Predicting amino acid changes that affect protein function. Nucleic Acids Res. *31*, 3812–3814.
94. Auton, A., Brooks, L.D., Durbin, R.M., Garrison, E.P., Kang, H.M., Korbel, J.O., Marchini, J.L., McCarthy, S., McVean, G.A., Abecasis, G.R.; and 1000 Genomes Project Consortium (2015). A global reference for human genetic variation. Nature *526*, 68–74.
95. Kircher, M., Witten, D.M., Jain, P., O'Roak, B.J., Cooper, G.M., and Shendure, J. (2014). A general framework for estimating the relative pathogenicity of human genetic variants. Nat. Genet. *46*, 310–315.
96. Boyko, A.R., Quignon, P., Li, L., Schoenebeck, J.J., Degenhardt, J.D., Lohmueller, K.E., Zhao, K., Brisbin, A., Parker, H.G., vonHoldt, B.M., et al. (2010). A simple genetic architecture underlies morphological variation in dogs. PLoS Biol. *8*, e1000451.
97. Wiener, P., Sánchez-Molano, E., Clements, D.N., Woolliams, J.A., Haskell, M.J., and Blott, S.C. (2017). Genomic data illuminates demography, genetic structure and selection of a popular dog breed. BMC Genomics *18*, 609.
98. Lohmueller, K.E. (2014). The distribution of deleterious genetic variation in human populations. Curr. Opin. Genet. Dev. *29*, 139–146.
99. Mezzavilla, M., Vozzi, D., Badii, R., Alkowari, M.K., Abdulhadi, K., Girotto, G., and Gasparini, P. (2015). Increased rate of deleterious variants in long runs of homozygosity of an inbred population from Qatar. Hum. Hered. *79*, 14–19.
100. Mooney, J.A., Huber, C.D., Service, S., Sul, J.H., Marsden, C.D., Zhang, Z., Sabatti, C., Ruiz-Linares, A., Bedoya, G., Freimer, N., Lohmueller, K.E.; and Costa Rica/Colombia Consortium for Genetic Investigation of Bipolar Endophenotypes (2018). Understanding the Hidden Complexity of Latin American Population Isolates. Am. J. Hum. Genet. *103*, 707–726.

The American Journal of Human Genetics, Volume *105*

Supplemental Data

Ancestry-Dependent Enrichment of Deleterious Homozygotes in Runs of Homozygosity

Zachary A. Szpiech, Angel C.Y. Mak, Marquitta J. White, Donglei Hu, Celeste Eng, Esteban G. Burchard, and Ryan D. Hernandez

Supplemental Data

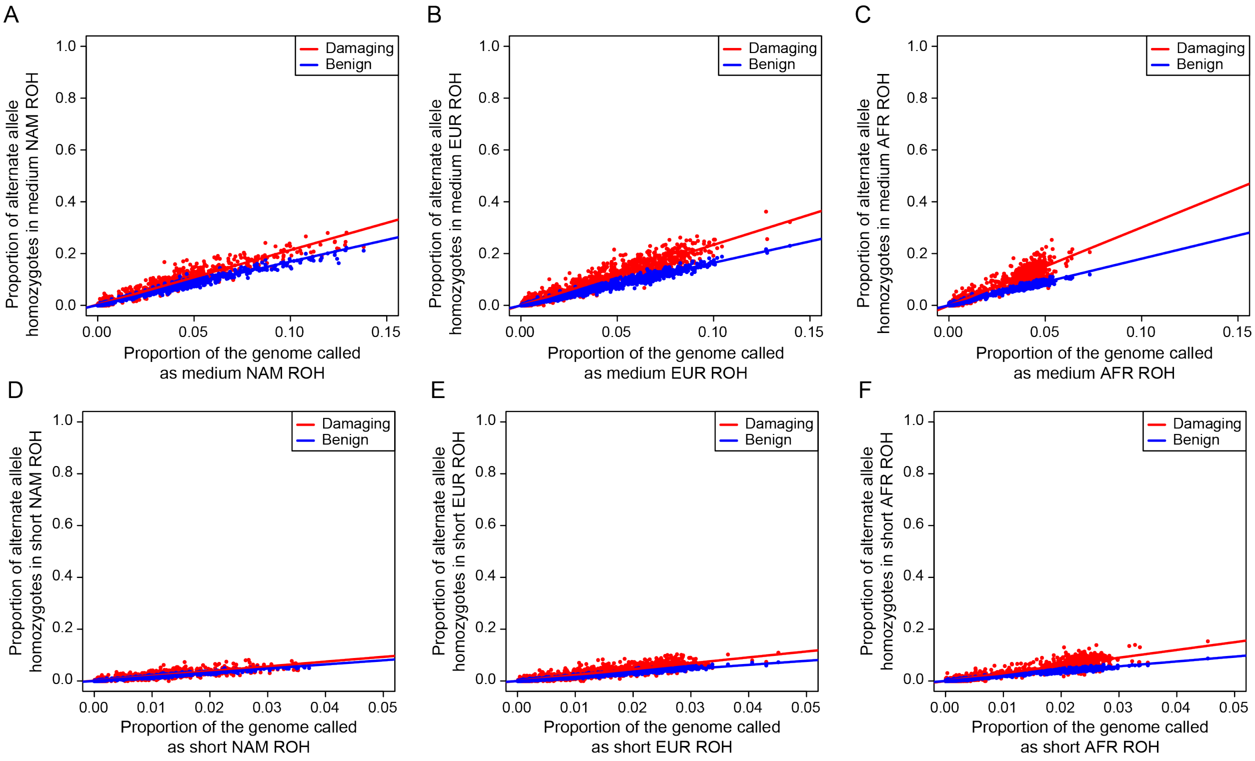

Figure S1. Deleterious versus benign homozygotes by ancestry for medium and small ROH.

The proportion of damaging (red) and benign (blue) homozygotes falling in ROH comprised of different ancestral haplotypes and size classes: (A) medium NAM ROH, (B) medium EUR ROH, (C) medium AFR ROH, (D) short NAM ROH, (E) short EUR ROH, and (F) short AFR ROH. EUR – European, AFR – African, and NAM – Native American.

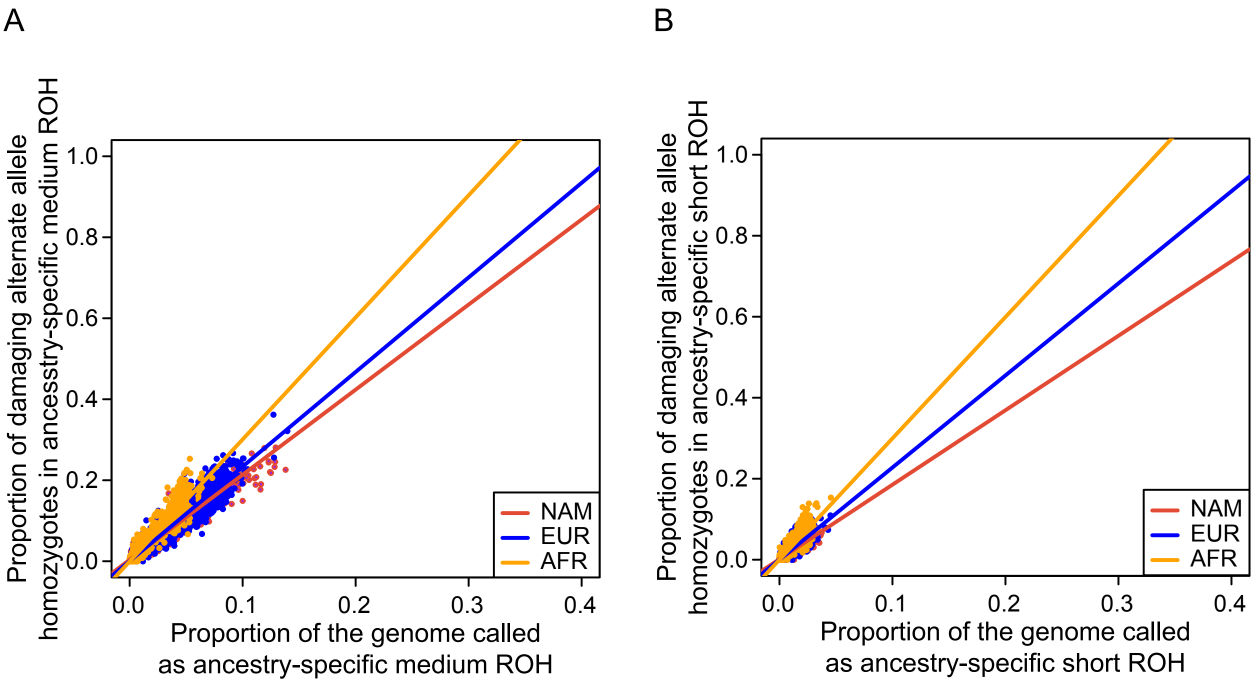

Figure S2. Comparison of deleterious homozygotes between ancestries for medium and small ROH.

A direct comparison of the proportion of damaging homozygotes falling in ROH comprised of different ancestral haplotypes for (A) medium ROH and (B) short ROH. EUR – European, colored blue; AFR – African, colored orange; and NAM – Native American, colored red.

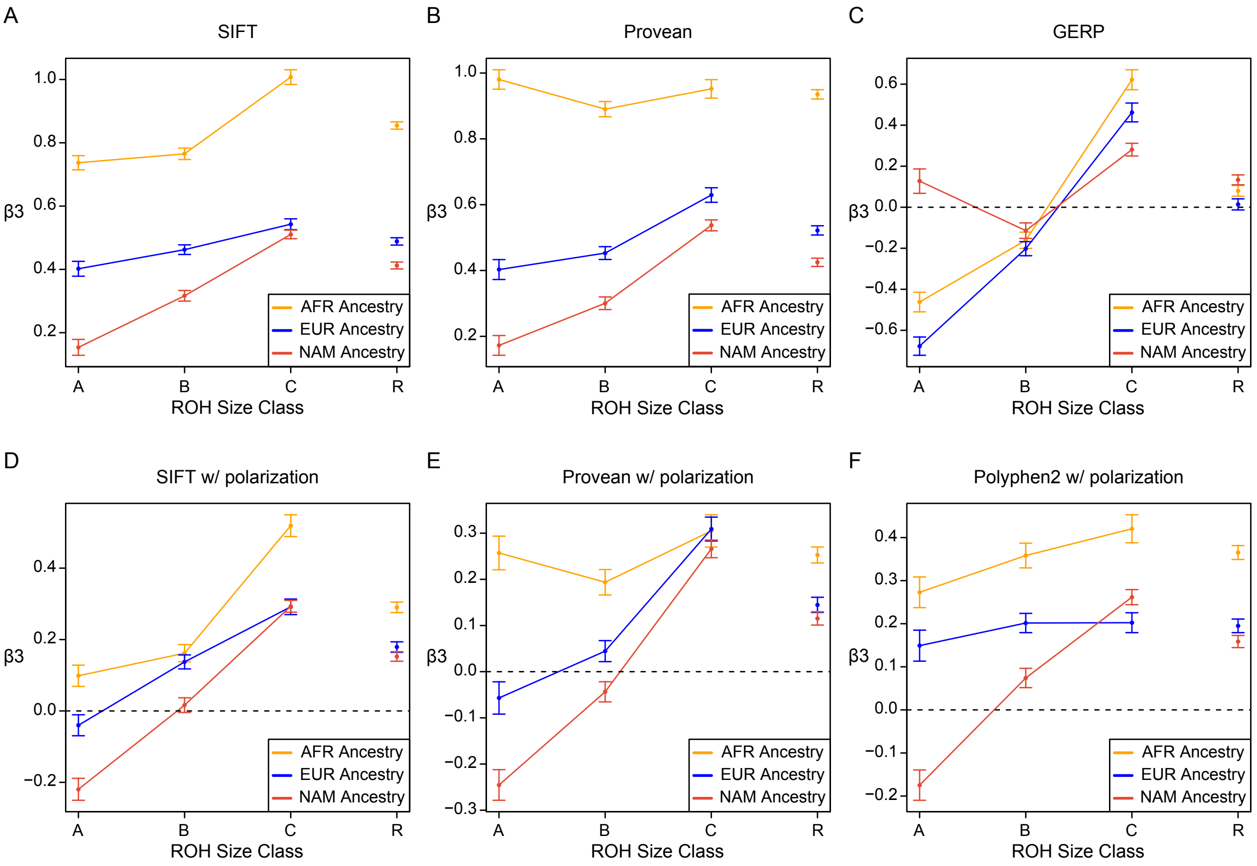

Figure S3. Regression coefficients for analyses with other deleteriousness classifications.

The difference in slopes (β_3 coefficients) between deleterious and benign categories across ROH size classes from re-analyses of the data using different deleteriousness classification schemes. (A) SIFT, (B) Provean, (C) GERP, (D) SIFT only with derived alleles, (E) Provean only with derived alleles, (F) Polyphen 2 only with derived alleles. ROH size classes: A – short, B – medium, C – long, R – all sizes. EUR – European, colored blue; AFR – African, colored orange; and NAM – Native American, colored red.

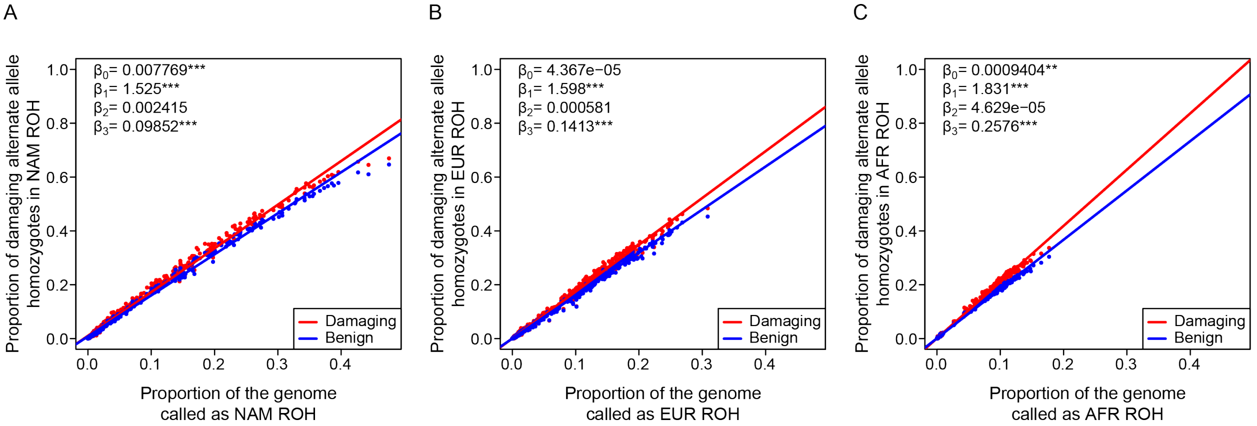

Figure S4. Replication of findings using 1000 genomes data.
The identical analysis from Fig 4A-C except using the six admixed populations from the 1000 Genomes Project and CADD scores. (A) Native American ancestry, (B) European Ancestry, (C) African Ancestry.

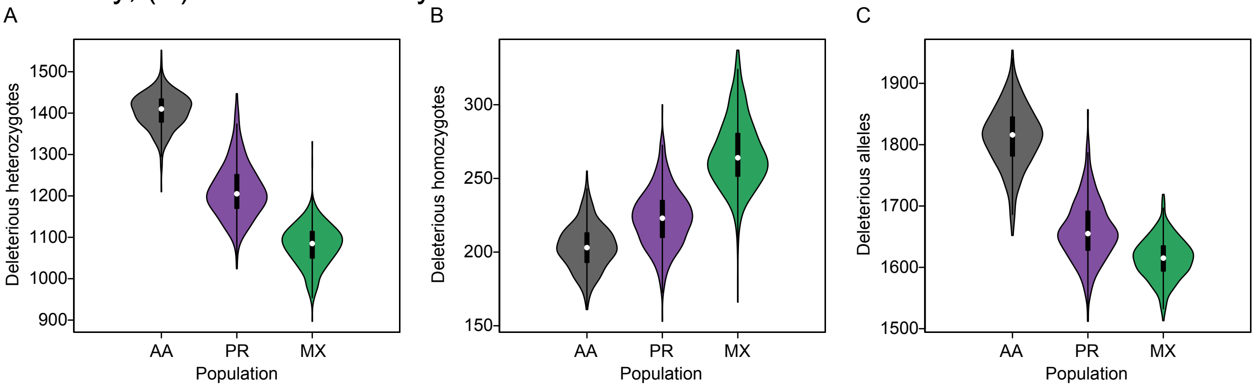

Figure S5. The distribution of polarized Polyphen2 deleterious alleles across populations.
The number of (A) deleterious heterozygotes, (B) deleterious homozygotes, and (C) total deleterious alleles per individual using polarized Polyphen2 classifications. AA – African American, PR – Puerto Rican, MX – Mexican American.

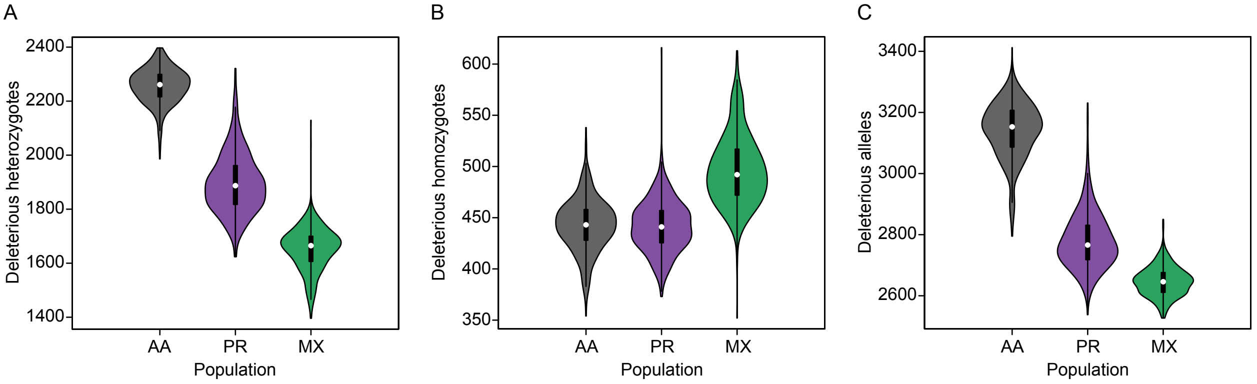

Figure S6. The distribution of SIFT deleterious alleles across populations.
The number of (A) deleterious heterozygotes, (B) deleterious homozygotes, and (C) total deleterious alleles per individual using SIFT classifications. AA – African American, PR – Puerto Rican, MX – Mexican American.

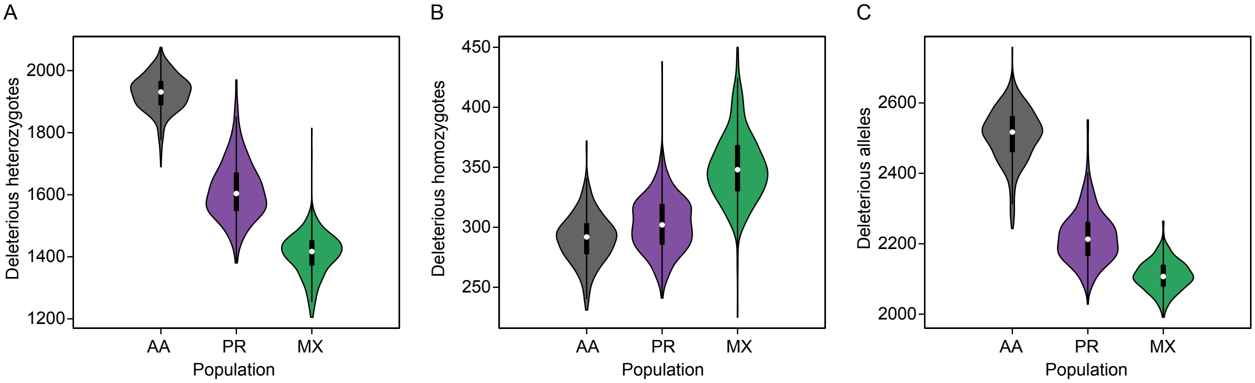

Figure S7. The distribution of polarized SIFT deleterious alleles across populations.
The number of (A) deleterious heterozygotes, (B) deleterious homozygotes, and (C) total deleterious alleles per individual using polarized SIFT classifications. AA – African American, PR – Puerto Rican, MX – Mexican American.

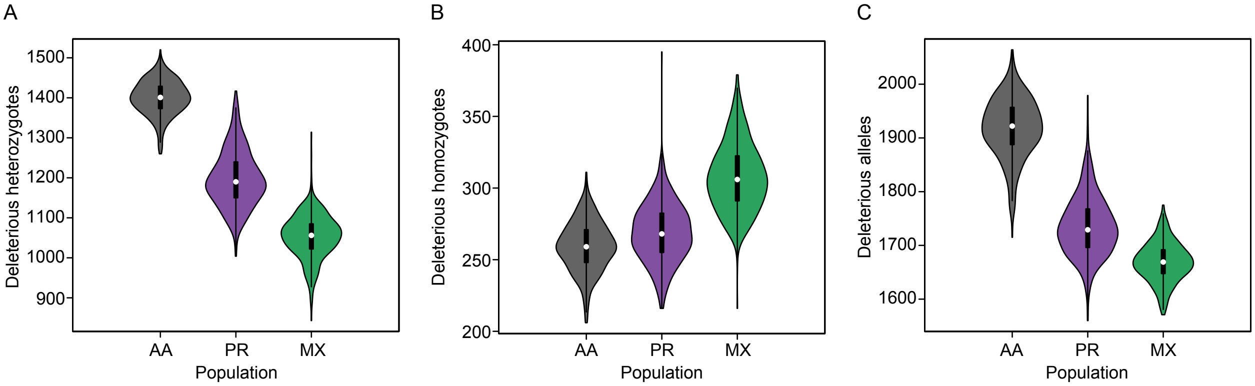

Figure S8. The distribution of Provean deleterious alleles across populations.
The number of (A) deleterious heterozygotes, (B) deleterious homozygotes, and (C) total deleterious alleles per individual using Provean classifications. AA – African American, PR – Puerto Rican, MX – Mexican American.

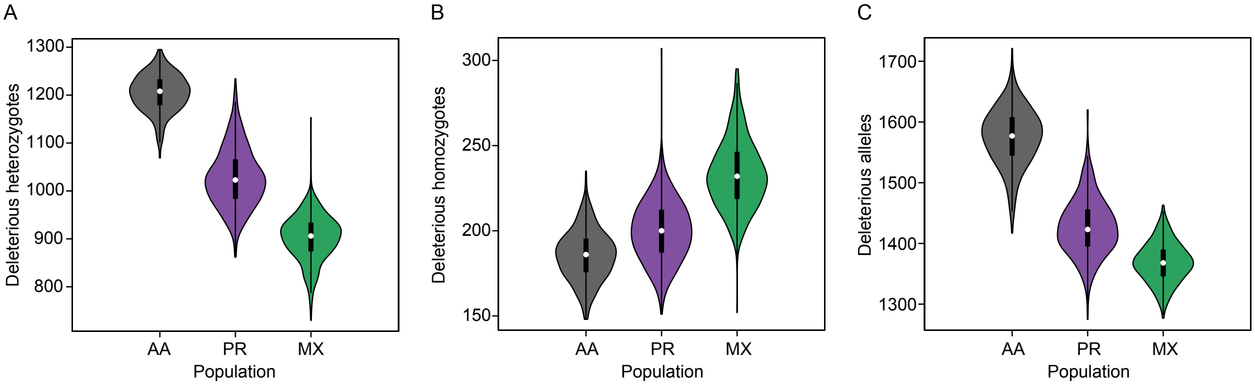

Figure S9. The distribution of polarized Provean deleterious alleles across populations.
The number of (A) deleterious heterozygotes, (B) deleterious homozygotes, and (C) total deleterious alleles per individual using polarized Provean classifications. AA – African American, PR – Puerto Rican, MX – Mexican American.

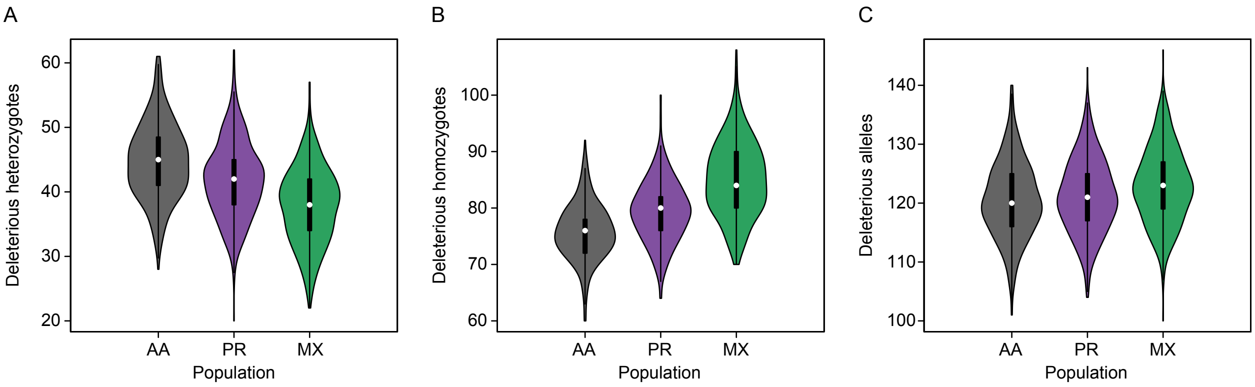

Figure S10. The distribution of GERP deleterious alleles across populations. The number of (A) deleterious heterozygotes, (B) deleterious homozygotes, and (C) total deleterious alleles per individual using GERP classifications. AA – African American, PR – Puerto Rican, MX – Mexican American.

Table S1. Regression coefficients inferred for the analyses shown in Figure 4 with p-values in parentheses.

ROH Class	β_0 (p-value)	β_1 (p-value)	β_2 (p-value)	β_3 (p-value)
All	***3.122×10^{-2} ($< 2 \times 10^{-16}$)	***1.460 ($< 2 \times 10^{-16}$)	***0.180 ($< 2 \times 10^{-16}$)	1.807×10^{-2} (0.0671)
Long	1.059×10^{-3} (0.508)	***1.429 ($< 2 \times 10^{-16}$)	***5.335×10^{-2} ($< 2 \times 10^{-16}$)	***0.229 ($< 2 \times 10^{-16}$)
Medium	***8.510×10^{-3} ($< 3.86 \times 10^{-5}$)	***1.584 ($< 2 \times 10^{-16}$)	***7.012×10^{-2} ($< 2 \times 10^{-16}$)	*5.695×10^{-2} (0.0173)
Short	***1.265×10^{-2} (4.61×10^{-7})	***1.424 ($< 2 \times 10^{-16}$)	***4.810×10^{-2} ($< 2 \times 10^{-16}$)	***−0.428 (1.10×10^{-8})

* $p < 0.05$, ** $p < 0.01$, *** $p < 0.001$.

Table S2. Regression coefficients inferred for the analyses shown in Figure 5 and Figure S1 with p-values in parentheses.

ROH Class	Ancestral Haplotype	β_0 (p-value)	β_1 (p-value)	β_2 (p-value)	β_3 (p-value)
All	NAM	$^{***}3.455 \times 10^{-3}$ (3.18×10^{-4})	$^{***}1.594$ $(< 2 \times 10^{-16})$	$^{***}7.369 \times 10^{-3}$ (5.87×10^{-8})	$^{***}0.510$ $(< 2 \times 10^{-16})$
	EUR	-1.205×10^{-3} (0.2081)	$^{***}1.546$ $(< 2 \times 10^{-16})$	2.497×10^{-3} (6.52×10^{-2})	$^{***}0.648$ $(< 2 \times 10^{-16})$
	AFR	$^{*}1.308 \times 10^{-3}$ (3.11×10^{-2})	$^{***}1.743$ $(< 2 \times 10^{-16})$	-1.210×10^{-3} (0.1587)	$^{***}1.214$ $(< 2 \times 10^{-16})$
Long	NAM	$^{**}1.989 \times 10^{-3}$ (3.38×10^{-3})	$^{***}1.545$ $(< 2 \times 10^{-16})$	$^{***}4.192 \times 10^{-3}$ (1.28×10^{-5})	$^{***}0.604$ $(< 2 \times 10^{-16})$
	EUR	-3.026×10^{-4} (0.623)	$^{***}1.403$ $(< 2 \times 10^{-16})$	1.260×10^{-3} (0.148)	$^{***}0.624$ $(< 2 \times 10^{-16})$
	AFR	5.324×10^{-4} (0.286)	$^{***}1.610$ $(< 2 \times 10^{-16})$	1.134×10^{-4} (0.872)	$^{***}1.265$ $(< 2 \times 10^{-16})$
Medium	NAM	$^{*}1.237 \times 10^{-3}$ (1.661×10^{-2})	$^{***}1.679$ $(< 2 \times 10^{-16})$	$^{**}2.325 \times 10^{-3}$ (1.46×10^{-3})	$^{***}0.421$ $(< 2 \times 10^{-16})$
	EUR	-5.614×10^{-4} (0.340)	$^{***}1.651$ $(< 2 \times 10^{-16})$	8.918×10^{-4} (0.284)	$^{***}0.684$ $(< 2 \times 10^{-16})$
	AFR	4.961×10^{-4} (0.246)	$^{***}1.797$ $(< 2 \times 10^{-16})$	-1.001×10^{-3} (0.0979)	$^{***}1.217$ $(< 2 \times 10^{-16})$
Short	NAM	2.711×10^{-4} (0.259)	$^{***}1.594$ $(< 2 \times 10^{-16})$	$^{**}1.073 \times 10^{-3}$ (1.61×10^{-3})	$^{***}0.244$ (1.27×10^{-13})
	EUR	-1.125×10^{-4} (0.720)	$^{***}1.665$ $(< 2 \times 10^{-16})$	4.023×10^{-4} (0.364)	$^{***}0.609$ $(< 2 \times 10^{-16})$
	AFR	2.349×10^{-4} (0.430)	$^{***}1.881$ $(< 2 \times 10^{-16})$	-2.568×10^{-4} (0.542)	$^{***}1.116$ $(< 2 \times 10^{-16})$

* $p < 0.05$, ** $p < 0.01$, *** $p < 0.001$.

Table S3. Regression coefficients inferred for the analyses shown in Figure 6 and Figure S2 with p-values in parentheses.

ROH Class	β_0 (p-value)	β_1 (p-value)	β_2 (p-value)	β_3 (p-value)	β_4 (p-value)	β_5 (p-value)
All	9.861×10^{-5} (0.922)	***2.957 ($< 2 \times 10^{-16}$)	1.193×10^{-3} (0.431)	***-1.072×10^{-2} (1.87×10^{-12})	***-0.763 ($< 2 \times 10^{-16}$)	***-0.852 ($< 2 \times 10^{-16}$)
Long	5.002×10^{-4} (0.449)	***2.878 ($< 2 \times 10^{-16}$)	4.477×10^{-4} (0.661)	***5.453×10^{-3} (7.11×10^{-8})	***-0.852 ($< 2 \times 10^{-16}$)	***-0.727 ($< 2 \times 10^{-16}$)
Medium	-4.804×10^{-4} (0.443)	***3.013 ($< 2 \times 10^{-16}$)	8.098×10^{-4} (0.387)	***4.002×10^{-3} (2.38×10^{-5})	***-0.678 ($< 2 \times 10^{-16}$)	***-0.912 ($< 2 \times 10^{-16}$)
Short	-2.196×10^{-5} (0.953)	***2.996 ($< 2 \times 10^{-16}$)	3.114×10^{-4} (0.571)	*1.351×10^{-3} (0.0149)	***-0.723 ($< 2 \times 10^{-16}$)	***-1.158 ($< 2 \times 10^{-16}$)

* p < 0.05, ** p < 0.01, *** p < 0.001.

Table S4. Regression coefficients inferred for the analyses comparing deleterious homozygotes in high-pLI versus low-pLI gene regions with p-values in parentheses.

ROH Class	β_0 (p-value)	β_1 (p-value)	β_2 (p-value)	β_3 (p-value)
All	***0.209 ($< 2 \times 10^{-16}$)	***1.463 ($< 2 \times 10^{-16}$)	***-9.924×10^{-5} (0.992)	*7.243×10^{-2} (0.0253)
Long	***5.280×10^{-2} ($< 2 \times 10^{-16}$)	***1.637 ($< 2 \times 10^{-16}$)	3.482×10^{-3} (0.634)	9.639×10^{-2} (0.0960)
Medium	***7.894×10^{-2} ($< 2 \times 10^{-16}$)	***1.641 ($< 2 \times 10^{-16}$)	5.712×10^{-2} (0.552)	-2.262×10^{-2} (0.774)
Short	***5.869×10^{-2} (3.10×10^{-13})	***0.975 (8.89×10^{-9})	-2.103×10^{-2} (0.0636)	*0.502 (0.0359)

* p < 0.05, ** p < 0.01, *** p < 0.001.

Table S5. Regression coefficients inferred for the analysis of deleterious homozygotes in high-pLI versus low-pLI genes with p-values in parentheses.

ROH Class	Ancestral Haplotype	β_0 (p-value)	β_1 (p-value)	β_2 (p-value)	β_3 (p-value)
All	NAM	***1.048×10^{-2} (2.54×10^{-6})	***2.087 ($< 2 \times 10^{-16}$)	1.065×10^{-3} (0.735)	**0.0746 (9.030×10^{-3})
	EUR	4.995×10^{-4} (0.849)	***2.160 ($< 2 \times 10^{-16}$)	6.582×10^{-3} (0.0767)	0.0584 (0.114)
	AFR	4.160×10^{-4} (0.828)	***2.947 ($< 2 \times 10^{-16}$)	-3.183×10^{-3} (0.239)	-8.246×10^{-3} (0.852)
Long	NAM	***6.190×10^{-3} (6.360×10^{-4})	***2.134 ($< 2 \times 10^{-16}$)	3.357×10^{-4} (0.896)	*0.0973 (1.571×10^{-2})
	EUR	7.646×10^{-4} (0.680)	***1.963 ($< 2 \times 10^{-16}$)	1.824×10^{-3} (0.487)	*0.133 (2.520×10^{-2})
	AFR	6.344×10^{-4} (0.709)	***2.882 ($< 2 \times 10^{-16}$)	-2.579×10^{-4} (0.915)	0.146 (0.122)
Medium	NAM	*3.217×10^{-3} (3.730×10^{-2})	***2.087 ($< 2 \times 10^{-16}$)	2.060×10^{-3} (0.346)	0.0595 (0.313)
	EUR	-2.446×10^{-4} (0.893)	***2.347 ($< 2 \times 10^{-16}$)	3.787×10^{-3} (0.140)	-0.0894 (0.136)
	AFR	4.961×10^{-4} (0.825)	***3.020 ($< 2 \times 10^{-16}$)	-1.287×10^{-3} (0.552)	0.0922 (0.288)
Short	NAM	1.299×10^{-3} (0.0528)	***1.786 ($< 2 \times 10^{-16}$)	-8.920×10^{-4} (0.347)	-0.0725 (0.427)
	EUR	3.451×10^{-4} (0.763)	***2.194 ($< 2 \times 10^{-16}$)	5.937×10^{-4} (0.714)	**0.304 (8.350×10^{-3})
	AFR	1.858×10^{-4} (0.851)	***2.923 ($< 2 \times 10^{-16}$)	-1.299×10^{-3} (0.354)	***-0.496 (2.260×10^{-6})

* $p < 0.05$, ** $p < 0.01$, *** $p < 0.001$.

Table S6. Regression coefficients for the analysis of deleterious homozygotes in high-pLI genes compared across ancestry backgrounds with p-values in parentheses.

ROH Class	β_0 (p-value)	β_1 (p-value)	β_2 (p-value)	β_3 (p-value)	β_4 (p-value)	β_5 (p-value)
All	-2.767×10^{-3} (0.312)	***2.938 ($< 2 \times 10^{-16}$)	*9.848×10^{-3} (1.747×10^{-2})	***1.431×10^{-2} (5.580×10^{-4})	***−0.720 ($< 2 \times 10^{-16}$)	***−0.777 ($< 2 \times 10^{-16}$)
Long	2.916×10^{-4} (0.883)	***3.030 ($< 2 \times 10^{-16}$)	2.271×10^{-3} (0.458)	*5.994×10^{-3} (4.790×10^{-2})	***−0.934 ($< 2 \times 10^{-16}$)	***−0.796 ($< 2 \times 10^{-16}$)
Medium	-1.545×10^{-3} (0.442)	***3.110 ($< 2 \times 10^{-16}$)	5.076 (0.0904)	*6.762×10^{-3} (2.570×10^{-2})	***−0.852 ($< 2 \times 10^{-16}$)	***−0.962 ($< 2 \times 10^{-16}$)
Short	-1.113×10^{-3} (0.368)	***2.427 ($< 2 \times 10^{-16}$)	2.051×10^{-3} (0.266)	1.515×10^{-3} (0.415)	0.071 (0.597)	***−0.713 (1.270×10^{-5})

* p < 0.05, ** p < 0.01, *** p < 0.001.

Table S7. Regression coefficients for the meta-analysis of simulation results with p-values in parentheses.

ROH Class	β_0 (p-value)	β_1 (p-value)	β_2 (p-value)	β_3 (p-value)	β_4 (p-value)	β_5 (p-value)
All	***5.781×10^{-2} ($< 2 \times 10^{-16}$)	***1.687 ($< 2 \times 10^{-16}$)	***4.209×10^{-2} ($< 2 \times 10^{-16}$)	***5.209×10^{-2} ($< 2 \times 10^{-16}$)	***−0.409 ($< 2 \times 10^{-16}$)	***−0.488 ($< 2 \times 10^{-16}$)
Long	***1.657×10^{-2} ($< 2 \times 10^{-16}$)	***1.907 ($< 2 \times 10^{-16}$)	***3.277×10^{-3} (3.950×10^{-7})	***4.892×10^{-3} (3.645×10^{-12})	***−0.386 ($< 2 \times 10^{-16}$)	***−0.446 ($< 2 \times 10^{-16}$)
Medium	***5.049×10^{-3} (6.983×10^{-7})	***1.976 ($< 2 \times 10^{-16}$)	**4.819×10^{-3} (3.246×10^{-3})	***6.103×10^{-3} (2.979×10^{-4})	***−0.390 ($< 2 \times 10^{-16}$)	***−0.473 ($< 2 \times 10^{-16}$)
Short	*2.731×10^{-3} (0.0247)	***1.969 ($< 2 \times 10^{-16}$)	2.497×10^{-3} (0.151)	-2.329×10^{-3} (0.182)	***−0.402 ($< 2 \times 10^{-16}$)	***−0.411 ($< 2 \times 10^{-16}$)

* p < 0.05, ** p < 0.01, *** p < 0.001.

Table S8. Top 0.1% of genes with high ROH coverage.

Gene Symbol	Description	AA	MX	PR
AC006486.9	Uncharacterized	X		
ALDOC	Aldolase, Fructose-Bisphosphate C			X
ALG2	ALG2 Alpha-1,3/1,6-Mannosyltransferase	X		
AMDHD2	Amidohydrolase Domain Containing 2		X	
ANAPC11	Anaphase Promoting Complex Subunit 11			X
ASNA1	ArsA Arsenite Transporter, ATP-Binding, Homolog 1 (Bacterial)		X	
BSDC1	BSD Domain Containing 1		X	
CCDC189	Coiled-Coil Domain Containing 189	X	X	X
CEMP1	Cementum Protein 1		X	
CFHR3	Complement Factor H Related 3	X		
CIC	Capicua Transcriptional Repressor	X		
CTD-2144E22.5	Uncharacterized			X
EDF1	Endothelial Differentiation Related Factor 1	X		
ERF	ETS2 Repressor Factor	X		
ERLIN2	ER Lipid Raft Associated 2			X
FAM229A	Family With Sequence Similarity 229 Member A		X	
GSTK1	Glutathione S-Transferase Kappa 1			X
GSTT1	Glutathione S-Transferase Theta 1	X		
HDAC1	Histone Deacetylase 1		X	
KCTD3	Potassium Channel Tetramerization Domain Containing 3			X
KHDRBS1	KH RNA Binding Domain Containing, Signal Transduction Associated 1		X	
MAFG	MAF BZIP Transcription Factor G			X
MAL	Mal, T Cell Differentiation Protein	X		
MAMDC4	MAM Domain Containing 4	X		
MARCKSL1	MARCKS Like 1		X	
NLK	Nemo Like Kinase		X	X
NPB	Neuropeptide B			X
PCYT2	Phosphate Cytidylyltransferase 2, Ethanolamine			X
PDCD7	Programmed Cell Death 7	X	X	X
PHKG2	Phosphorylase Kinase Catalytic Subunit Gamma 2	X	X	X
PHPT1	Phosphohistidine Phosphatase 1	X		
PIGV	Phosphatidylinositol Glycan Anchor Biosynthesis Class V	X		
PLBD2	Phospholipase B Domain Containing 2	X		
PROSC	Pyridoxal Phosphate Binding Protein			X
PRSS27	Serine Protease 27		X	
RP11-863K10.7	Uncharacterized			X
SEC61B	Sec61 Translocon Beta Subunit	X		
SIRT7	Sirtuin 7			X
SPAG5	Sperm Associated Antigen 5		X	X
STARD7	StAR Related Lipid Transfer Domain Containing 7	X		
TMEM139	Transmembrane Protein 139	X	X	X
TRIR	Telomerase RNA Component Interacting RNase		X	
TSSK3	Testis Specific Serine Kinase 3		X	
ZNF703	Zinc Finger Protein 703	X		X

Table S9. Linear regression of total ROH length in megabases onto total local ancestry length in megabases by population.

	β_0 (p-value)	β_1 (p-value)	β_2 (p-value)
African American	$^{***}4.446 \times 10^{+2}$ ($< 2 \times 10^{-16}$)	$^{***}0.0784$ ($< 2 \times 10^{-16}$)	$^{***}0.0863$ ($< 2 \times 10^{-16}$)
Puerto Rican	$^{***}2.798 \times 10^{+2}$ ($< 2 \times 10^{-16}$)	$^{***}0.292$ ($< 2 \times 10^{-16}$)	$^{***}0.258$ ($< 2 \times 10^{-16}$)
Mexican American	-37.276 (0.291)	$^{***}0.3670$ ($< 2 \times 10^{-16}$)	$^{***}0.491$ ($< 2 \times 10^{-16}$)

β_0 is the regression coefficient for the intercept. β_1 is the regression coefficient for European Ancestry. β_2 is the regression coefficient for Native American ancestry. * p < 0.05, ** p < 0.01, *** p < 0.001.

Table S10. Gene regions with runs of homozygosity significantly enriched for European ancestry in Puerto Rican individuals.

See file TableS10.xlsx.

Table S11. Gene regions with runs of homozygosity significantly enriched for African ancestry in Puerto Rican American individuals.

chr:pos	Gene Symbol	p-value
chr6:30103885-30116512	*TRIM40*	8.57E-11
chr6:32361740-32374905	*BTNL2*	2.22E-10
chr6:29555683-29556745	*OR2H2*	6.79E-10
chr6:29364416-29365448	*OR12D2*	1.32E-09
chr6:28048753-28057341	*ZNF165*	2.71E-08
chr6:31514647-31526606	*NFKBIL1*	3.65E-07
chr6:31548302-31550299	*LTB*	7.20E-07
chr6:29523406-29601753	*GABBR1*	1.06E-06
chr6:31543344-31546113	*TNF*	1.13E-06
chr6:31539831-31542101	*LTA*	1.55E-06

Table S12. Gene regions with runs of homozygosity significantly enriched for Native American ancestry in Puerto Rican American individuals.

chr:pos	Gene Symbol	p-value
chr14:57671888-57673291	*AL391152.1*	4.90E-08
chr14:57735627-57756797	*AP5M1*	9.43E-08
chr14:57670518-57735726	*EXOC5*	1.45E-07
chr22:39348746-39359188	*APOBEC3A*	7.85E-07
chr2:54480315-54483409	*TSPYL6*	9.71E-07
chr14:57857262-57882635	*NAA30*	1.01E-06

chr9:140098534-140100090	*TMEM203*	2.44E-06

Table S13. Gene regions with runs of homozygosity significantly enriched for European ancestry in Mexican American individuals.

chr:pos	name	p-value
chr2:111271389-111334762	*RGPD6*	2.22E-12
chr15:48431625-48470714	*MYEF2*	1.71E-09
chr15:48413169-48434869	*SLC24A5*	1.78E-09
chr15:28623767-28637170	*GOLGA8F*	1.54E-07
chr15:42450899-42500514	*VPS39*	2.32E-07
chr1:248651885-248652898	*OR2T5*	4.18E-07
chr15:69745123-69748255	*RPLP1*	1.39E-06
chr17:34639793-34641846	*CCL4L2*	2.07E-06

Table S14. Gene regions with runs of homozygosity significantly enriched for African ancestry in Mexican American individuals.

chr:pos	Gene Symbol	p-value
chr6:30103885-30116512	*TRIM40*	2.50E-18
chr6:30585486-30594172	*MRPS18B*	6.25E-13
chr6:32361740-32374905	*BTNL2*	2.84E-12
chr6:30509154-30524951	*GNL1*	4.02E-12
chr6:30539153-30564956	*ABCF1*	1.60E-11
chr6:27106073-27114619	*HIST1H2BK*	1.84E-11
chr6:27107076-27108418	*HIST1H4I*	2.32E-11
chr6:30568177-30586389	*PPP1R10*	2.56E-11
chr6:26538633-26546482	*HMGN4*	3.92E-11
chr6:30524663-30531500	*PRR3*	1.58E-10
chr6:27093676-27100541	*HIST1H2BJ*	1.59E-10
chr6:30594619-30614600	*ATAT1*	1.75E-10
chr6:27100832-27103070	*HIST1H2AG*	1.94E-10
chr6:30667584-30685666	*MDC1*	2.76E-10
chr6:31865562-31913449	*C2*	3.37E-10
chr6:30152232-30181204	*TRIM26*	5.62E-10
chr6:30119722-30128711	*TRIM10*	8.15E-10
chr6:30644166-30655672	*PPP1R18*	1.42E-09
chr6:30614816-30620987	*C6orf136*	2.46E-09
chr6:30130993-30140473	*TRIM15*	2.57E-09
chr6:29555683-29556745	*OR2H2*	3.19E-09
chr6:27342394-27371683	*ZNF391*	3.34E-09
chr6:29364416-29365448	*OR12D2*	7.44E-09
chr6:29393281-29424848	*OR11A1*	1.04E-08
chr6:26501449-26510650	*BTN1A1*	1.09E-08

chr6:30655824-30659197	*NRM*	1.45E-08
chr6:29523406-29601753	*GABBR1*	1.96E-08
chr6:30457244-30461982	*HLA-E*	3.16E-08
chr6:27114861-27115317	*HIST1H2AH*	3.65E-08
chr6:31895475-31919825	*CFB*	4.71E-08
chr6:31895475-31919861	*CFB*	4.74E-08
chr6:31783291-31785723	*HSPA1A*	5.47E-08
chr6:30620896-30640814	*DHX16*	6.55E-08
chr6:31777396-31783437	*HSPA1L*	7.16E-08
chr6:32407619-32412823	*HLA-DRA*	7.38E-08
chr6:29407083-29408731	*OR10C1*	1.39E-07
chr6:26597180-26600278	*ABT1*	2.03E-07
chr6:26281283-26285762	*HIST1H4H*	2.14E-07
chr6:26225383-26225844	*HIST1H3E*	2.30E-07
chr6:31919864-31926887	*NELFE*	2.47E-07
chr6:26183958-26184454	*HIST1H2BE*	3.12E-07
chr6:26158349-26171577	*HIST1H2BD*	4.06E-07
chr6:26188938-26189304	*HIST1H4D*	4.10E-07
chr6:27418522-27440897	*ZNF184*	4.15E-07
chr6:26234440-26235216	*HIST1H1D*	5.55E-07
chr6:31795512-31798031	*HSPA1B*	5.59E-07
chr6:26217165-26217711	*HIST1H2AE*	6.30E-07
chr6:29323007-29399744	*OR5V1*	7.03E-07
chr6:26216428-26216872	*HIST1H2BG*	8.07E-07
chr6:26273144-26273622	*HIST1H2BI*	1.03E-06
chr6:29640169-29648887	*ZFP57*	1.39E-06
chr6:26240561-26240976	*HIST1H4F*	1.59E-06
chr6:26271146-26271612	*HIST1H3G*	1.71E-06
chr6:32485120-32498064	*HLA-DRB5*	2.47E-06

Table S15. Gene regions with runs of homozygosity significantly enriched for Native American ancestry in Mexican American individuals.

See file TableS15.xlsx.

Table S16. Gene regions with runs of homozygosity significantly enriched for European ancestry in African American individuals.

chr:pos	Gene Symbol	p-value
chr1:248651885-248652898	*OR2T5*	3.80E-10
chr1:248636627-248637634	*OR2T3*	3.24E-09
chr1:248721784-248722797	*OR2T29*	8.18E-09
chr1:248813232-248814185	*OR2T27*	3.33E-08
chr9:125512019-125513062	*OR1L6*	5.28E-08
chr20:39314488-39317880	*MAFB*	1.26E-07
chr13:64320934-64321323	*AL445989.1*	1.75E-07
chr6:3224495-3231964	*TUBB2B*	1.76E-07
chr20:45947246-45949467	*AL031666.2*	1.85E-07
chr1:68511645-68517314	*DIRAS3*	2.46E-07
chr1:248684916-248685964	*OR2G6*	3.07E-07
chr21:46031996-46032871	*KRTAP10-8*	3.10E-07
chr1:248616077-248617130	*OR2T2*	3.86E-07
chr17:61678231-61685725	*TACO1*	5.14E-07
chr9:125551150-125552237	*OR5C1*	1.05E-06
chr7:27282164-27290112	*EVX1*	1.06E-06
chr10:695888-711109	*PRR26*	1.10E-06
chr1:36023074-36032875	*NCDN*	1.18E-06
chr9:125437315-125438432	*OR1L3*	1.23E-06
chr14:105952654-105955284	*CRIP1*	1.37E-06
chr17:18427880-18430160	*FAM106A*	1.61E-06
chr1:248801588-248802559	*OR2T35*	1.68E-06
chr1:248185250-248186188	*OR2L5*	1.69E-06
chr3:128779610-128781249	*GP9*	1.74E-06
chr1:248201474-248202607	*OR2L2*	2.07E-06
chr4:71554196-71556267	*UTP3*	2.52E-06

Table S17. Gene regions with runs of homozygosity significantly enriched for African ancestry in African American individuals.

chr:pos	Gene Symbol	p-value
chr3:87276421-87304698	*CHMP2B*	4.12E-08
chr12:123745528-123756881	*CDK2AP1*	2.72E-07

Supplemental Methods

Processing of 1000 genomes data. Pemberton and Szpiech [1] published an analysis of deleterious variation and ROH in over 2,500 individuals from the 1000 Genomes Project [2] spanning the world. Included among these were individuals from six different population groups known to have non-trivial amounts of admixture ($n = 503$). In order to replicate our results, we obtained all the data from Pemberton and Szpiech [1] and extracted only the individuals from each of these six admixed populations populations: African American (ASW, n = 61), Afro-Caribbean (ACB, n = 95), Columbian (CLM, n = 94), Puerto Rican (PUR, n = 104), Peruvian (PEL, n = 85), and Mexican American (MXL, n = 64).

In order to call local ancestry tracts, we generated three putative ancestral populations using data from two sources. First, we obtained Affymetrix 6.0 genotyping data for both European (CEU, n = 99) and Yoruban (YRI, n = 108) individuals available from the 1000 Genomes Project [2]. Next, we obtained genotype data (n = 350) from the Native Mexican Diversity Panel (NMDP) [3], which were also typed on Affymetrix 6.0. The NMDP samples may have non-native admixture in them, and therefore we need to identify and exclude those with non-trivial amounts of inferred non-native ancestry from our reference panel. After merging these three data sets, we phase with SHAPEIT2 [4], and then we do an ADMIXTURE [5] analysis for k = 3 components and n = 20 replications, under the assumption that two of these components would identify European and African ancestry and the remaining component would be Native American. From the full set of data, we then filter anyone with < 99.9% ancestry from any given component. This leaves us with 86 CEU, 106 YRI, and 111 NMDP individuals, which we used as reference populations for RFMix [6] local ancestry inference of our 503 admixed samples.

ROH calls for each of these individuals were also extracted from the full set of data accompanying Pemberton and Szpiech [1], which were originally generated by Blant, et al. [7].

Deleteriousness information and counts were also obtained from Pemberton and Szpiech [1]. In their analysis, they used CADD scores [8]. CADD scores assign a numerical value to a mutation, with higher number suggesting more deleteriousness. For our analyses here, we combined all mutations with CADD $>= 15$ into the “damaging” category and all mutations with CADD $<= 15$ into the “benign” category.

We then reran the analysis described in the main text, finding qualitatively similar results (Figure S4).

1. Pemberton, T.J., and Szpiech, Z.A. (2018). Relationship between Deleterious Variation, Genomic Autozygosity, and Disease Risk: Insights from The 1000 Genomes Project. Am J Hum Genet 102, 658-675.
2. Genomes Project, C., Auton, A., Brooks, L.D., Durbin, R.M., Garrison, E.P., Kang, H.M., Korbel, J.O., Marchini, J.L., McCarthy, S., McVean, G.A., et al. (2015). A global reference for human genetic variation. Nature 526, 68-74.
3. Moreno-Estrada, A., Gignoux, C.R., Fernandez-Lopez, J.C., Zakharia, F., Sikora, M., Contreras, A.V., Acuna-Alonzo, V., Sandoval, K., Eng, C., Romero-Hidalgo, S., et al. (2014). Human genetics. The genetics of Mexico recapitulates Native American substructure and affects biomedical traits. Science 344, 1280-1285.
4. Delaneau, O., Zagury, J.F., and Marchini, J. (2013). Improved whole-chromosome phasing for disease and population genetic studies. Nat Methods 10, 5-6.
5. Alexander, D.H., Novembre, J., and Lange, K. (2009). Fast model-based estimation of ancestry in unrelated individuals. Genome Res 19, 1655-1664.

6. Maples, B.K., Gravel, S., Kenny, E.E., and Bustamante, C.D. (2013). RFMix: a discriminative modeling approach for rapid and robust local-ancestry inference. Am J Hum Genet 93, 278-288.
7. Blant, A., Kwong, M., Szpiech, Z.A., and Pemberton, T.J. (2017). Weighted likelihood inference of genomic autozygosity patterns in dense genotype data. BMC Genomics 18, 928.
8. Kircher, M., Witten, D.M., Jain, P., O'Roak, B.J., Cooper, G.M., and Shendure, J. (2014). A general framework for estimating the relative pathogenicity of human genetic variants. Nat Genet 46, 310-315.

Supplemental Acknowledgements

The “NHLBI TOPMed: Genes-environments and Admixture in Latino Asthmatics (GALA II) Study” was supported by NIH and NHLBI grant R01HL117004; study enrollment supported by NIEHS grant R01ES015794, the Sandler Family Foundation, the American Asthma Foundation, the RWJF Amos Medical Faculty Development Program, Harry Wm. and Diana V. Hind Distinguished Professor in Pharmaceutical Sciences II. All study collaborators: Shannon Thyne, UCSF; Harold J. Farber, Texas Children's Hospital; Denise Serebrisky, Jacobi Medical Center; Rajesh Kumar, Lurie Children's Hospital of Chicago; Emerita Brigino-Buenaventura, Kaiser Permanente; Michael A. LeNoir, Bay Area Pediatrics; Kelley Meade, Children's Hospital, Oakland; William Rodriguez-Cintron, VA Hospital, Puerto Rico; Pedro C. Avila, Northwestern University, Jose R. Rodriguez-Santana, Centro de Neumologia Pediatrica. The authors acknowledge the families and patients for their participation and thank the numerous health care providers and community clinics for their support and participation in GALA II. In particular, the authors thank study coordinator Sandra Salazar; the recruiters who obtained the data: Duanny Alva, MD, Gaby Ayala-Rodriguez, Lisa Caine, Elizabeth Castellanos, Jaime Colon, Denise DeJesus, Blanca Lopez, Brenda Lopez, MD, Louis Martos, Vivian Medina, Juana Olivo, Mario Peralta, Esther Pomares, MD, Jihan Quraishi, Johanna Rodriguez, Shahdad Saeedi, Dean Soto, Ana Taveras. See publication: PMID: 23750510.

The “NHLBI TOPMed: Study of African Americans, Asthma, Genes and Environment (SAGE)” was supported by NIH and NHLBI grants R01HL117004 and X01HL134589; study enrollment supported by the Sandler Family Foundation, the American Asthma Foundation, the RWJF Amos Medical Faculty Development Program, Harry Wm. and Diana V. Hind Distinguished Professor in Pharmaceutical Sciences II.
